# A demographic model for sex ratio evolution and the effects of sex‐biased offspring costs

**DOI:** 10.1002/ece3.1902

**Published:** 2016-02-09

**Authors:** Esther Shyu, Hal Caswell

**Affiliations:** ^1^ Biology Department MS‐34Woods Hole Oceanographic InstitutionWoods HoleMA02543USA; ^2^ Institute for Biodiversity and Ecosystem DynamicsUniversity of AmsterdamAmsterdamThe Netherlands

**Keywords:** adaptive dynamics, evolutionarily singular strategies, matrix population models, offspring costs, reproductive value, sex ratio evolution, two‐sex models

## Abstract

The evolution of the primary sex ratio, the proportion of male births in an individual's offspring production strategy, is a frequency‐dependent process that selects against the more common sex. Because reproduction is shaped by the entire life cycle, sex ratio theory would benefit from explicitly two‐sex models that include some form of life cycle structure. We present a demographic approach to sex ratio evolution that combines adaptive dynamics with nonlinear matrix population models. We also determine the evolutionary and convergence stability of singular strategies using matrix calculus. These methods allow the incorporation of any population structure, including multiple sexes and stages, into evolutionary projections. Using this framework, we compare how four different interpretations of sex‐biased offspring costs affect sex ratio evolution. We find that demographic differences affect evolutionary outcomes and that, contrary to prior belief, sex‐biased mortality after parental investment can bias the primary sex ratio (but not the corresponding reproductive value ratio). These results differ qualitatively from the widely held conclusions of previous models that neglect demographic structure.

## Introduction

Sex ratio evolution is the one of the oldest life‐history questions and a well‐known example of frequency‐dependent selection. Although the primary sex ratio *s*_1_ (proportion of offspring that are born male) is nearly equal in many mammals, including humans, sex ratio biases have been observed in countless other species (Karlin and Lessard 1986). Explanations for biased sex ratios often focus on demographic differences (e.g., costs of offspring, mortality of specific life cycle stages); however, much sex ratio theory is based on purely verbal arguments or models with minimal demographic structure.

Early explanations of sex ratio evolution, for instance, relied on occasionally confused or vague verbal reasoning. Darwin (1871) wrote that parents producing more of the rarer sex would have fewer superfluous offspring and thus be more “productive,” but later admitted the problem was too intricate for him to reason through (Darwin 1874). Fisher (1930) tackled this challenge with a famously succinct, and infamously cryptic, verbal argument based on reproductive value (the present value of an individual's future offspring). Because every individual has a male and female parent, Fisher stated that the “total reproductive value” of each sex in a given generation (i.e., their genetic contributions to all future generations, West 2009) must be equal.

If males and females are equally costly to produce, the sex ratio should evolve to equality, as any sex produced in excess will have fewer mating opportunities, less reproductive success, and thus smaller returns on reproductive value; parents who are genetically predisposed to producing the rarer sex thus have more grandchildren to propagate their genes, making the once rarer sex more common over time (Hamilton 1967; West 2009). If, however, males and females are differentially costly (e.g., require different amounts of resources to produce), Fisher claimed the sex ratio will evolve so that there is equal “expenditure” in, rather than equal numbers of, both sexes.

Trivers (1972) more precisely defined this expenditure as “parental investment” – any investment a parent makes (time, energy, resources, protection, etc.) to increase an offspring's survival and reproductive success, at the cost of investing in other children. If a son, for example, requires less parental investment than a daughter, a parent can produce more successful sons (and, to a point, more reproductive value) per unit investment. Selection thus biases the sex ratio toward sons until there is equal parental investment in sons and daughters. The optimal primary sex ratio $s_{1}^{*}$ is given by the “equal investment principle”:

(1) $$\begin{array}{cc} C_{m} & s_{1}^{*}=\;C_{f}\left(1-s_{1}^{}*\right)\\ & s_{1}^{*}=\;\frac{C_{f}}{C_{m}+C_{f}} \end{array}$$

where *C*_*m*_ and *C*_*f*_ are some form of male and female investment costs (Charnov 1982; Hardy 2002). Others (e.g., Charnov 1982; Bull and Charnov 1988; Frank 1990) have shown that the equal investment principle (1) requires several implicit assumptions, including random mating, fixed resource allocation, and additive offspring costs with linear returns (e.g., doubling your investment in sons doubles the grandchildren or genetic returns that your sons produce).

Early mathematical treatments of Darwin and Fisher's arguments by Düsing (1883, translated in Edwards 2000) and Shaw and Mohler (1953) are the basis for many other sex ratio analyses. They consider how an individual's sex ratio affects their fitness, through their relative number of (or genetic contribution to) grandchildren. The fitness *w* of a given parent has the form:

(2) $$w=\frac{n}{4N}\left(\frac{s_{1}}{S_{1}}+\frac{1-s_{1}}{1-S_{1}}\right)$$

when that parent produces *n* offspring at a primary sex ratio *s*_1_, and the population at large produces *N* offspring at a primary sex ratio *S*_1_. This formulation does not consider stage structure within the sexes, nor does it account for offspring production over more than two generations.

The fitness of a given sex ratio phenotype *s*_1_ is frequency‐dependent, in that it depends on the population sex ratio *S*_1_. When the population sex ratio *S*_1_ = 0.5, (2) is always $w=\frac{n}{2N}$, regardless of the individual sex ratio *s*_1_ (Shaw and Mohler 1953); this means that all sex ratios, including the resident and any mutants, will have the same fitness. Thus, when *S*_1_ = 0.5, no individual sex ratio can have greater fitness than the resident, so no alternatives sex ratios can increase under selection. The equal sex ratio *S*_1_ = 0.5 is thus an “unbeatable” evolutionarily stable strategy (ESS) (Maynard Smith and Price 1973). Subsequent studies have identified numerous factors that can bias the ESS sex ratio from 0.5, including local mate competition (Hamilton 1967), maternal condition (Trivers and Willard 1973), parent–offspring conflict (Trivers 1974; Trivers and Hare 1976), and other unusual life history strategies or sex determination systems (Hardy 2002).

The ESS sex ratios can be affected by sex‐biased offspring costs, especially in terms of parental investment and the timing of sex‐biased mortality (Hardy 2002; West 2009). Shaw and Mohler (1953) noted that sex‐specific survival probabilities cancel out of (2) and are thus irrelevant to selection, although they did not consider parental investment. Fisher (1930) himself argued that only sex‐biased mortality *during* the period of parental investment affects the sex ratio, and later analyses have largely ruled in favor of his conjecture. Similar to Shaw and Mohler, some frame sex ratio fitness in terms of a genetic contribution to grandchildren (Kolman 1960). Others use a population genetics approach to track the allele frequencies of different sex ratios (Leigh 1970). The general consensus is that sex‐biased mortality *after* parental investment cannot bias the ESS sex ratio, because increased mortality is then compensated for by increased reproductive opportunities (West 2009).

However, few of the models underlying sex ratio evolution theory explicitly consider stage structure, even though the effect of mortality at different life cycle stages is an inherently demographic issue. While some models include age structure (e.g., Emlen 1968a,b; Charnov 1975; Charlesworth 1977), only a handful are capable of including more general stage structure, such as size, developmental stage, or parental quality (e.g., Leimar 1996; Schindler et al. 2015). Our models can incorporate all three levels of structure, with age structure appearing as a special case of stage structure.

The interaction between the sexes, and the effects of distorted adult sex ratios on the mating success of males and females, requires nonlinear models. Most models in the literature, even those that incorporate population structure (e.g., Pen et al. 1999; Fawcett et al. 2011), assume that reproduction is unaffected by adult sex ratio. This removes one of the ways in which changes to the primary sex ratio may affect invasion fitness.

Many sex ratio models also only consider offspring production over two generations, rather than over an entire lifetime. Models that incorporate both age structure and lifetime offspring production are rarer and have produced more mixed results. In some age‐structured models (Charnov 1975), sex‐biased survival cancels out of the fitness expression, while in others (Emlen 1968a,b), mortality at all reproducing ages affects the sex ratio. These discrepancies suggest that stage‐specific, demographic factors deserve additional consideration in sex ratio theory.

Here, we use matrix population models to incorporate multiple sexes, stages, and life cycle events into our evolutionary projections. Furthermore, although previous studies of sex ratio evolution (e.g., Charnov 1979a, 1982; Hardy 2002; Otto and Day 2007) have focused on finding ESSs, additional methods are needed to determine whether the population will actually converge to the ESS in the long run. Here, we apply adaptive dynamics to identify potential evolutionary outcomes and characterize both their evolutionary and convergence stability.

## Model and Methods

There are two main approaches for studying sex ratio evolution (West 2009). One approach uses population or quantitative genetics to track the dynamics of allele frequencies (e.g., Eshel 1975; Charlesworth 1977; Uyenoyama and Bengtsson 1979; Karlin and Lessard 1986) . The other approach, which includes ESS theory and adaptive dynamics, ignores the often complex underlying genetics and instead focuses on trait phenotypes (e.g., Charnov 1982; Hardy 2002; Otto and Day 2007). We will use the latter approach by considering population‐level effects of the sex ratio phenotype.

Following the two‐sex modeling framework introduced in E. Shyu and H. Caswell (in review a) , we construct a series of continuous‐time rate matrices that incorporate multiple sexes, stages, and life cycle events. Because these models are frequency‐dependent, their long‐term population growth rates are given by the dominant eigenvalue of the projection matrix at the equilibrium stage distribution (Caswell and Weeks 1986; Pollak 1986; Hadeler et al. 1988; Iannelli et al. 2005) . By applying adaptive dynamics theory, we use these models to identify and characterize long‐term evolutionary outcomes for the primary sex ratio – namely, singular strategies including, but not limited, to ESSs.

### The two‐sex matrix model

**Figure 1:**
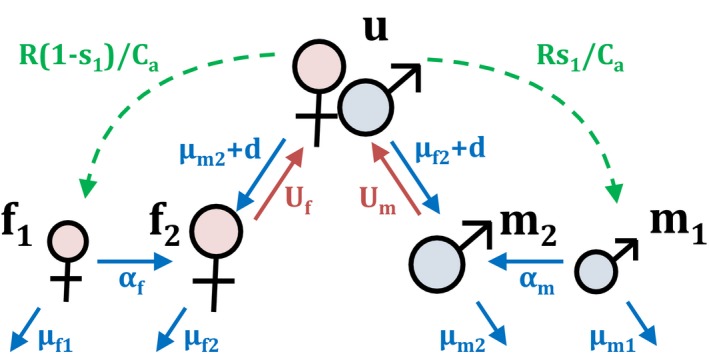
Life cycle diagram for a 5‐stage population with juvenile males *m*_1_ and juvenile females *f*_1_, adult males *m*_2_ and adult females *f*_2_, and reproducing unions *u*. The functions and parameters shown here appear in the union formation matrix **U** (6) (red), birth matrix **B** (7) (green), or transition matrix **T** (8) (blue) (from E. Shyu and H. Caswell in review a).

Consider a population with five stages: juvenile males *m*_1_ and juvenile females *f*_1_, adult males *m*_2_ and adult females *f*_2_, and reproducing unions *u* (mated couples with one male and one female each). Single adults interact to form unions, which then produce new juvenile offspring (Fig. 1). A summary of the variables, parameters, and matrices in this model is provided in Table 1.

**Table 1:** A summary of the variables, parameters, matrices, and population properties in the two‐sex matrix model. Mutant parameters (not shown) are denoted by an apostrophe; for example, **A**′ is the mutant projection matrix

**Matrices and Vectors**	
**A**	projection matrix (9)
**B**	birth matrix (7)
**U**	union formation matrix (6)
**T**	transition matrix (8)
**n**	population density vector (3)
**p**	population frequency vector (12)
$\hat{p}$ or **w**	equilibrium stage structure
**v**	reproductive value vector
**Population Properties**	
*λ*	long‐term population growth rate, dominant eigenvalue of $A(\hat{p})$
*m*_1_, *m*_2_	juvenile, adult male stages
*f*_1_, *f*_2_	juvenile, adult female stages
*u*	union (pair) stage
*s*_1_	primary sex ratio (proportion of offspring that are born male)
$s_{1}^{*}$	singular strategy (SS) value of *s*_1_
*s*_2_	secondary sex ratio (proportion of adults that are male)
$s_{2}^{*}$	resulting *s*_2_ when $s_{1}=s_{1}^{*}$
*v*_*i*_	reproductive value of stage *i*
**Life Cycle Parameters**	
*α*_*m*_, *α*_*f*_	male, female maturation rates
*d*	divorce rate (rate at which a male‐female pair bond breaks)
*μ*_*f*1_, *μ*_*f*2_	juvenile, adult female mortality rates
*μ*_*m*1_, *μ*_*m*2_	juvenile, adult male mortality rates
*R*	resource investment rate
*M*	mating function (4) (total unions formed per time)
*U*_*m*_, *U*_*f*_	per capita mating rates (5)
*F*_*m*_, *F*_*f*_	per capita fertility rates (63)
**Offspring Cost Parameters**	
*C*_*m*_, *C*_*f*_	male, female offspring resource costs
*C*_*a*_	average offspring resource cost (35)
*a*	age of independence
*I*	offspring investment rate
*D*_*m*_, *D*_*f*_	male, female parental mortality costs
*E*_*m*_, *E*_*f*_	male, female parent costs of reproduction (42)
*μ*_*m*2*c*_, *μ*_*f*2*c*_	mated male, female mortality rates (44)

The population vector at time *t* is:

(3) $$n\left(t\right)=\left(\begin{array}{c} m_{1}\\ m_{2}\\ f_{1}\\ f_{2}\\ u \end{array}\right)$$

The total unions (pairs) formed per time is given by the nonlinear harmonic mean mating function:

(4) $$M\left(n\right)=\frac{2m_{2}f_{2}}{m_{2}+f_{2}}$$

which has frequency‐dependent male and female per capita mating rates:

(5) $$\begin{array}{cc} U_{m}\left(n\right)=\;\frac{M(n)}{m_{2}}\;=\;\frac{2f_{2}}{m_{2}+f_{2}} & \\ U_{f}\left(n\right)=\;\frac{M(n)}{f_{2}}\;=\;\frac{2m_{2}}{m_{2}+f_{2}} \end{array}$$

Mating, birth, and life cycle transition processes are divided into three rate matrices (**U**,** B**, and **T**) as follows.

1. The union formation matrix **U** contains the per capita mating rates (5):
  (6) $$U\left(n\right)=\left(\begin{array}{ccccc} 0 & 0 & 0 & 0 & 0\\ 0 & -U_{m}\left(p\right) & 0 & 0 & 0\\ 0 & 0 & 0 & 0 & 0\\ 0 & 0 & 0 & -U_{f}\left(p\right) & 0\\ 0 & \frac{1}{2}U_{m}\left(p\right) & 0 & \frac{1}{2}U_{f}\left(p\right) & 0 \end{array}\right)$$
2. The birth matrix **B** contains the rates of male and female offspring production by unions:
  (7) $$B=\left(\begin{array}{ccccc} 0 & 0 & 0 & 0 & \frac{Rs_{1}}{C_{a}}\\ 0 & 0 & 0 & 0 & 0\\ 0 & 0 & 0 & 0 & \frac{R(1-s_{1})}{C_{a}}\\ 0 & 0 & 0 & 0 & 0\\ 0 & 0 & 0 & 0 & 0 \end{array}\right)$$
  where *s*_1_ is the (evolving) primary sex ratio, *R* is the total resource investment rate, *C*_*a*_ is the average offspring resource cost per birth. The quantity *R*/*C*_*a*_ is the union reproductive rate (offspring produced per time).
3. The life cycle transition matrix **T** contains the rates of mortality and transitions between stages:
  (8) $$T=\left(\begin{array}{ccccc} -(\mu_{m1}+\alpha_{m}) & 0 & 0 & 0 & 0\\ \alpha_{m} & -\mu_{m2} & 0 & 0 & \mu_{f2}+d\\ 0 & 0 & -(\mu_{f1}+\alpha_{f}) & 0 & 0\\ 0 & 0 & \alpha_{f} & -\mu_{f2} & \mu_{m2}+d\\ 0 & 0 & 0 & 0 & -(\mu_{m2}+\mu_{f2}+d) \end{array}\right)$$
  where *μ*_*m*1_ and *μ*_*m*2_ are the juvenile and adult male mortality rates, *μ*_*f*1_ and *μ*_*f*2_ are the juvenile and adult female mortality rates, *α*_*m*_ and *α*_*f*_ are the male and female maturation rates, and *d* is the union divorce rate.

The average of these three rate matrices is the continuous‐time projection matrix

(9) $$A\left(n\right)=\frac{1}{3}\left[T+B+U(n)\right]$$

where

(10) $$\frac{dn}{dt}=A\left(n\right)n\left(t\right)$$

In our model, the nonlinear mating rates (5) are homogeneous of degree 0 with respect to **n**. This allows all entries *a*_*ij*_ in **A** to depend on relative stage frequencies rather than absolute abundances, that is:

(11) $$a_{ij}\left(cn\right)=a_{ij}\left(n\right)$$

for any positive constant *c*. As a result, population growth is frequency dependent, in that it is a function of the population frequency vector:

(12) $$p=\frac{n}{\left||n|\right|}$$

where ‖**n**‖ is the 1‐norm of **n**.

Frequency‐dependent models like these ultimately converge to an equilibrium stage distribution $\hat{p}$. The population then grows or decays exponentially at a rate given by the dominant eigenvalue *λ* of $A(\hat{p})$. For calculating *λ*, it is sufficient to consider the dynamics of **p** (E. Shyu and H. Caswell in review a):

(13) $$\frac{dp}{dt}=\left(I_{s}-p1^{⊺}\right)A\left(p\right)p$$

To find $\hat{p}$, integrate (13) with the MATLAB ODE45 differential equation solver until **p** converges to $\hat{p}$ (e.g., until vector entries do not change significantly over consecutive integration intervals). We then calculate the population's long‐term growth rate *λ*, the dominant eigenvalue of $A(\hat{p})$, and its corresponding right and left eigenvectors **w** and **v**. Note that the dominant right eigenvector of $A(\hat{p})$ equals the stable stage distribution; that is, $w=\hat{p}$.

### Evolutionary analysis with adaptive dynamics

Adaptive dynamics treats evolution as a series of “invasions” by mutant phenotypes. Mutations are assumed to occur infrequently, so that each mutation is either fixed or lost before the next mutation arises (Geritz et al. 1998). Because each mutant is initially rare, its effects on the existing resident population are considered negligible (Metz 2006).

Consider a stable, monomorphic resident population with phenotype *x*, projection matrix **A**, and growth rate *λ*. An invading mutant with phenotype *y*, projection matrix **A**′, and growth rate *λ*′ (which depends on the environmental conditions set by the resident) has two possible fates. If *λ*′ < *λ*, the mutant will ultimately die out. But if *λ*′ > *λ*, the mutant can replace the resident and induce evolutionary change (Metz et al. 1992).

#### The mutant projection matrix

Analogous to the resident projection matrix **A** in (9), the mutant projection matrix **A**′ is the average of the mutant rate matrices:

(14) $$A^{\prime }\left(\hat{p}\right)=\frac{1}{3}\left[T^{\prime }+B^{\prime }+U^{\prime }\left(\hat{p}\right)\right]$$

The only phenotypic difference between mutants and residents is the primary sex ratio they use. Just as the resident birth matrix **B** in (7) depends on the resident sex ratio *s*_1_, the mutant birth matrix **B**′ depends on mutant sex ratio $s_{1}^{\prime }$:

(15) $$B^{\prime }=\left(\begin{array}{ccccc} 0 & 0 & 0 & 0 & \frac{Rs_{1}^{\prime }}{C_{a}}\\ 0 & 0 & 0 & 0 & 0\\ 0 & 0 & 0 & 0 & \frac{R(1-s_{1}^{\prime })}{C_{a}}\\ 0 & 0 & 0 & 0 & 0\\ 0 & 0 & 0 & 0 & 0 \end{array}\right)$$

Because mutants are so rare, we assume they mate only with residents. As a result, the equilibrium resident population sets the overall mating rate according to (5), and the mutant mating matrix **U**′ is the resident mating matrix **U** in (6) evaluated at the resident stable stage distribution $\hat{p}$:

(16) $$U^{\prime }\left(\hat{p}\right)=\left(\begin{array}{ccccc} 0 & 0 & 0 & 0 & 0\\ 0 & -U_{m}\left(\hat{p}\right) & 0 & 0 & 0\\ 0 & 0 & 0 & 0 & 0\\ 0 & 0 & 0 & -U_{f}\left(\hat{p}\right) & 0\\ 0 & \frac{1}{2}U_{m}\left(\hat{p}\right) & 0 & \frac{1}{2}U_{f}\left(\hat{p}\right) & 0 \end{array}\right)$$

Unless certain transition rates also depend on the primary sex ratio (e.g., parental survival in “Case 4: parental mortality”, which has transition matrix (43)), the mutant transition matrix **T**′ is the same as the resident transition matrix **T** in (8).

#### Invasion fitness and the selection gradient

We define the invasion fitness *s*_*x*_(*y*) as the long‐term growth rate of a mutant with phenotype *y*, relative to the growth rate of a resident with phenotype *x*, in the equilibrium resident environment (as set by the resident's stable stage distribution $\hat{p}$). In our two‐sex matrix model, the invasion fitness is the difference in the dominant eigenvalues of the mutant and resident projection matrices (*λ* and *λ*′, respectively), where *λ*′ is evaluated at the resident's stable stage distribution $\hat{p}$.

(17) $$s_{x}\left(y\right)=\lambda^{\prime }\left(\hat{p}\right)-\lambda$$

Only mutants with a positive invasion fitness can displace the resident and cause evolutionary change.

The first derivative of the invasion fitness (17), with respect to the mutant phenotype *y*, is the selection gradient *D*(*x*), which indicates the direction of selection at a given resident phenotype *x*. In our model, the selection gradient is the sensitivity of mutant eigenvalue *λ*′ (Caswell 2010). In general, the resident and mutant phenotypes can be written as vectors of trait values, ***θ*** and ***θ***′, respectively. The selection gradient is then:

(18) $$\begin{array}{cc} D(x)= & \frac{\partial s_{x}\left(y\right)}{\partial y}{|}_{y=x}=\frac{\partial \lambda^{\prime }}{\partial \theta^{\prime ⊺}}{|}_{\theta^{\prime }=\theta }\\ = & (\left(w^{\prime ⊺}⊗v^{\prime ⊺}\right)\frac{d\text{vec}A^{\prime }}{d\theta^{\prime ⊺}}){|}_{\theta^{\prime }=\theta } \end{array}$$

where **w**′ and **v**′ are the dominant right and left eigenvectors of the mutant matrix $A^{\prime }\left(\hat{p}\right)$, scaled so that $v^{\prime ⊺}w^{\prime }=1$.

Here, we consider the case where the only evolving trait is the primary sex ratio. Thus, the trait vectors ***θ*** and ***θ***′ simplify to the scalar resident and mutant sex ratios *s*_1_ and $s_{1}^{\prime }$. The selection gradient at *s*_1_ is thus:

(19) $$\frac{\partial \lambda^{\prime }}{\partial s_{1}^{\prime }}{|}_{s_{1}^{\prime }=s_{1}}=\left(\left(w^{\prime ⊺}⊗v^{\prime ⊺}\right)\frac{d\text{vec}A^{\prime }}{ds_{1}^{\prime }}\right){|}_{s_{1}^{\prime }=s_{1}}$$

The selection gradient (19) can lend insight into both the transient and equilibrium evolutionary dynamics of *s*_1_. Although we will focus on equilibrium results here, transient evolutionary dynamics can also be explored using the canonical equation (as discussed in E. Shyu and H. Caswell in review b).

#### Singular strategies

When the selection gradient (19) is 0, there is no directional selection on *s*_1_. The corresponding resident strategy $s_{1}^{}*$ is called a singular strategy (SS). MATLAB's fsolve or fmincon functions can be used determine the values of *s*_1_ where the selection gradient vanishes, which correspond to $s_{1}^{}*$.

Singular strategies are potential long‐term evolutionary outcomes that can be characterized by several criteria (Geritz et al. 1998). One can, instance, determine whether each SS is evolutionarily stable (an ESS that is resistant to further invasion) or evolutionarily unstable (a branching point that leads to phenotypic divergence), as well as whether each SS is convergence stable (an evolutionary attractor that the population will converge to through small mutations) or convergence unstable (an evolutionary repeller).

**Figure 2:**
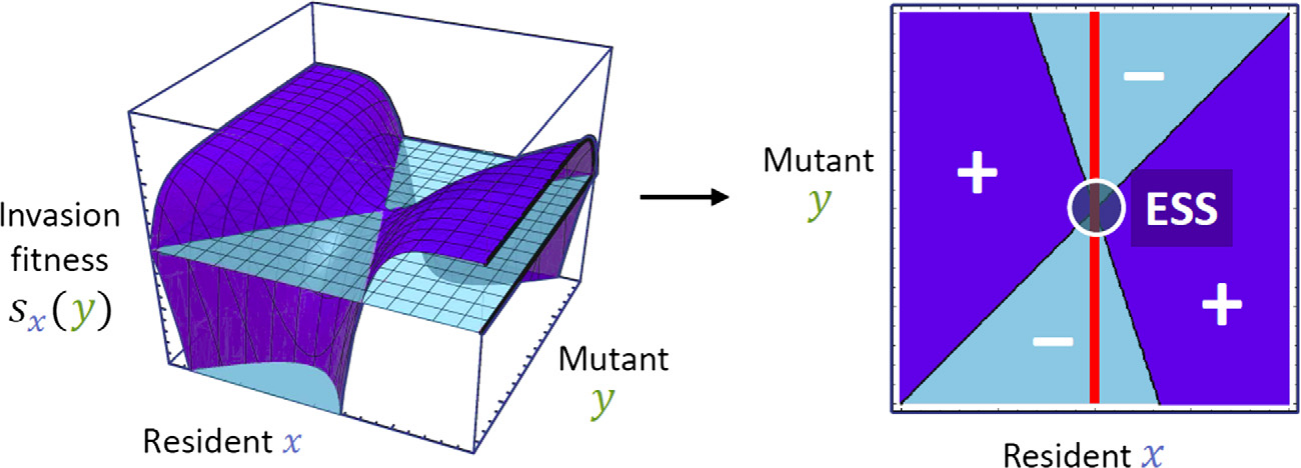
A 3D visualization of an invasion fitness landscape and the corresponding 2D pairwise invasion plot (PIP). The + on the PIP indicates where the invasion fitness is positive; the – indicates where the invasion fitness is negative.

For a one‐dimensional phenotype, 2D visualizations of the invasion fitness landscape called pairwise invasion plots (PIPs) graphically indicate evolutionary and convergence stability. A PIP shows where invasion fitness is positive or negative, depending on the resident phenotype *x* and the mutant phenotype *y* (Fig. 2).

**Figure 3:**
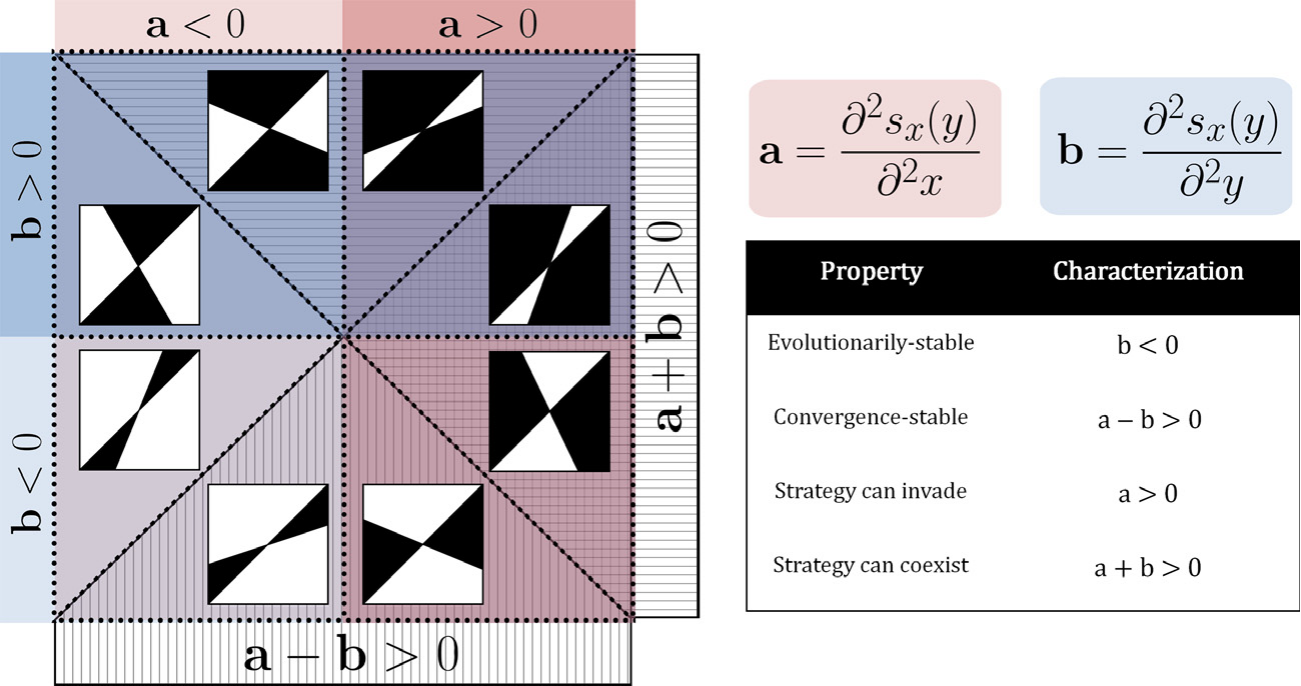
Second derivative properties and the corresponding pairwise invasion plots (PIPs) for the eight types of singular strategies (adapted from Geritz et al. 1998).

Singular strategies occur at intersections of the boundaries between negative and positive regions. If mutations are small (do not differ drastically from the resident phenotype), the behavior of the PIP around a SS indicates several properties (Fig. 3). If, for example, the vertical line through the SS is entirely in the negative region (as in Fig. 2), the SS is evolutionarily stable, that is, an ESS resistant to further invasion.

### Second derivatives of invasion fitness

Evolutionary and convergence stability can be determined more generally using the local second derivatives of the invasion fitness *s*_*x*_(*y*) (17) to the mutant phenotype *y* and the resident phenotype *x* (Fig. 3). In our two‐sex model, these are the second derivatives of the mutant and resident eigenvalues, *λ*′ and *λ*, with respect to the vectors describing mutant and resident phenotypes, ***θ***′ and ***θ***. Again, we will only consider the primary sex ratio phenotype, so ***θ*** and ***θ***′ simplify to *s*_1_ and $s_{1}^{\prime }$, respectively.

The pure second derivative with respect to the mutant phenotype is:

(20) $$\frac{\partial^{2}s_{x}\left(y\right)}{\partial^{2}y}\;=\;\frac{\partial^{2}\left(\lambda^{\prime }-\lambda \right)}{\partial \theta^{\prime }\partial \theta^{\prime ⊺}}\;=\;\frac{\partial^{2}\lambda^{\prime }}{\partial^{2}s_{1}^{\prime }}$$

because *λ* does not depend on the mutant sex ratio $s_{1}^{\prime }$.

The pure second derivative with respect to the resident phenotype is:

(21) $$\frac{\partial^{2}s_{x}\left(y\right)}{\partial^{2}x}\;=\;\frac{\partial^{2}\left(\lambda^{\prime }-\lambda \right)}{\partial \theta \partial \theta^{⊺}}\;=\;\frac{\partial^{2}\left(\lambda^{\prime }-\lambda \right)}{\partial^{2}s_{1}}$$

because both *λ*′ and *λ* depend on the resident sex ratio *s*_1_.

The evolutionary stability of a singular strategy x* depends on (20) (Geritz et al. 1998):

(22) $$\frac{\partial^{2}s_{x}\left(y\right)}{\partial^{2}y}{|}_{x=y=x^{}*}\left\{\begin{array}{cc} <\;0 & \text{evolutionarily stable (ESS)}\\ =0 & \text{may be selectively neutral (weak form ESS)}\\ >0 & \text{evolutionarily unstable (branch point)} \end{array}\right.$$

The convergence stability of a singular strategy x* depends on both (20) and (21) (Eshel 1983; Geritz et al. 1996):

(23) $$\left(\frac{\partial^{2}s_{x}\left(y\right)}{\partial^{2}x}-\frac{\partial^{2}s_{x}\left(y\right)}{\partial^{2}y}\right){|}_{x=y=x^{*}}\left\{\begin{array}{cc} >0 & \text{convergence stable (attracting)}\\ <0 & \text{convergence unstable (repelling)} \end{array}\right.$$

In next two sections, we will present matrix calculus equations for the pure second derivatives (20) and (21) that determine evolutionary and convergence stability. These expressions will rely on the equations (24) and (32) respectively.

#### Second derivatives with respect to the mutant sex ratio (20)

Calculating (20) requires the second derivatives of the mutant eigenvalue *λ*′ with respect to the mutant trait $s_{1}^{\prime }$. The corresponding mutant matrix **A**′ is a function of the mutant trait $s_{1}^{\prime }$ and the resident's stable stage structure $\hat{p}\left(s_{1}\right)$ (as mutants are rare, their environment is completely determined by the resident). Because $\hat{p}$ is constant, $A^{\prime }\left(s_{1}^{\prime },\hat{p}\right)$ is a constant matrix.

As shown in Shyu and Caswell (2014, (38)), the second derivatives of *λ*′ can be found using matrix calculus:

(24) $$\frac{\partial^{2}\lambda^{\prime }}{\partial^{2}s_{1}^{\prime }}=\left(w^{\prime ⊺}⊗v^{\prime ⊺}⊗I_{s}\right)H\left[\text{vec}A^{\prime };s_{1}^{\prime }\right]+{\left(\frac{d\text{vec}A^{\prime }}{ds_{1}^{\prime }}\right)}^{⊺}H\left[\lambda^{\prime };\text{vec}A^{\prime }\right]\frac{d\text{vec}A^{\prime }}{ds_{1}^{\prime }}$$

where ⊗ is the Kronecker product, vec is the vec operator, and **I**_*s*_ is a *s* × *s* identity matrix.

This expression depends on the Hessian (matrix of second derivatives) of *λ*′ with respect to **A**′:

(25a) $$H\left[\lambda^{\prime };\text{vec}A^{\prime }\right]=\frac{1}{2}\left(H_{1}+H_{1}^{⊺}\right)$$

where

(25b) $$H_{1}=\left(I_{n}⊗v^{\prime }\right)\frac{dw^{\prime }}{d{\text{vec}}^{⊺}A^{\prime }}+\left(w^{\prime }⊗I_{n}\right)\frac{dv^{\prime }}{d{\text{vec}}^{⊺}A^{\prime }}$$

and the first derivatives of **w**′ and **v**′ are

(26) $$\frac{dw^{\prime }}{d{\text{vec}}^{⊺}A^{\prime }}={\left(\lambda^{\prime }I_{n}-A^{\prime }+w^{\prime }e^{⊺}A^{\prime }\right)}^{-1}\left[w^{\prime ⊺}⊗\left(I_{n}-w^{\prime }e^{⊺}\right)\right]$$

(27) $$\begin{array}{cc} \frac{dv^{\prime }}{d{\text{vec}}^{⊺}A^{\prime }}= & \;\;{\left(\lambda^{\prime }I_{n}-A^{\prime ⊺}+\lambda^{\prime }v^{\prime }w^{\prime ⊺}\right)}^{-1}\\ & \times \left(\left(I_{n}-v^{\prime }w^{\prime ⊺}\right)⊗v^{\prime ⊺}-\lambda^{\prime }\left(v^{\prime }⊗v^{\prime ⊺}\right)\frac{dw^{\prime }}{d{\text{vec}}^{⊺}A^{\prime }}\right). \end{array}$$

where **e** ^⊺^ is a 1 × *s* vector of ones.

The expression (24) also depends on the first and second derivatives of **A**′ with respect to $s_{1}^{\prime }$, which are given by $\frac{d\text{vec}A^{\prime }}{ds_{1}^{\prime }}$ and $H[\text{vec}A^{\prime };s_{1}^{\prime }]$ respectively. Recall from (9) that:

(28) $$A^{\prime }=\frac{1}{3}\left(T^{\prime }+B^{\prime }+U^{\prime }\right)$$

The first derivatives of **A**′ to $s_{1}^{\prime }$ are:

(29) $$\frac{d\text{vec}A^{\prime }}{ds_{1}^{\prime }}=\frac{1}{3}\left(\frac{d\text{vec}T^{\prime }}{ds_{1}^{\prime }}+\frac{d\text{vec}B^{\prime }}{ds_{1}^{\prime }}+\frac{d\text{vec}U^{\prime }}{ds_{1}^{\prime }}\right)$$

The second derivatives of **A**′ to $s_{1}^{\prime }$ are:

(30) $$H\left[\text{vec}A^{\prime };s_{1}^{\prime }\right]=\frac{1}{3}\left(H\left[\text{vec}T^{\prime };s_{1}^{\prime }\right]+H\left[\text{vec}B^{\prime };s_{1}^{\prime }\right]+H\left[\text{vec}U^{\prime };s_{1}^{\prime }\right]\right)$$

These derivatives can be evaluated by hand or with a symbolic math program. Because not all of the matrices depend on $s_{1}^{\prime }$ (**U**′, for example, never does), both (29) and (30) may simplify considerably.

#### Second derivatives with respect to the resident sex ratio (21)

Calculating (21) requires the second derivatives of the mutant eigenvalue *λ*′ and resident eigenvalue *λ* with respect to the resident trait *s*_1_. The resident matrix **A** is a function of the resident trait *s*_1_ and the resident stable stage distribution $\hat{p}\left(s_{1}\right)$ (as mutants are rare, they do not affect resident dynamics). Because the resident's dynamics depend on its own stage distribution, $A(\hat{p})$ is a nonlinear, frequency‐dependent matrix.

Frequency dependence makes the second derivatives of *λ* difficult to calculate directly. But once (20) is found using (24), (21) can be calculated using the relationship:

(31) $$\left(\frac{\partial^{2}\lambda^{\prime }}{\partial^{2}s_{1}^{\prime }}+2\frac{\partial^{2}\lambda^{\prime }}{\partial s_{1}\partial s_{1}^{\prime }}+\frac{\partial^{2}\left(\lambda^{\prime }-\lambda \right)}{\partial^{2}s_{1}}\right){|}_{s_{1}^{\prime }=s_{1}=s_{1}^{}*}=0$$

which holds at any singular strategy $s_{1}^{}*$ (Appendix A).

The first term in (31) is given by (20). The second term in (31) is the mixed second derivatives of *λ*′ to $s_{1}^{\prime }$ and *s*_1_. This is shown in Appendix B to be:

(32) $$\frac{\partial^{2}\lambda^{\prime }}{\partial s_{1}\partial s_{1}^{\prime }}=\left(w^{\prime ⊺}⊗v^{\prime ⊺}⊗I_{s}\right)K_{n^{2},s}\frac{d\text{vec}C}{dw^{⊺}}\;\;\frac{dw}{ds_{1}}+C^{⊺}\left[\left(I_{n}⊗v^{\prime }\right)\frac{dw^{\prime }}{ds_{1}}+\left(w^{\prime }⊗I_{n}\right)\frac{dv^{\prime }}{ds_{1}}\right]$$

where

(33) $$C=\frac{d\text{vec}A^{\prime }}{ds_{1}^{\prime }}$$

Thus, at any singular strategy, (21) can be found by substituting the pure second derivative (20) and mixed second derivative (32) into the relationship (31). The convergence stability condition (23) thus becomes:

(34) $$\frac{\partial^{2}\lambda^{\prime }}{\partial^{2}s_{1}^{\prime }}+\frac{\partial^{2}\lambda^{\prime }}{\partial s_{1}\partial s_{1}^{\prime }}{|}_{x=y=x^{*}}\left\{\begin{array}{cc} <0 & \text{convergence stable (attracting)}\\ >0 & \text{convergence unstable (repelling)} \end{array}\right.$$

## Case Studies: Sex‐Biased Offspring Costs

**Figure 4:**
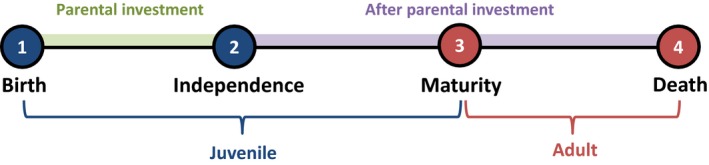
A timeline of key points in an individual's lifetime. The individual is a juvenile from Point 1 to 3, and an adult from Point 3 to 4. The period *during* parental investment is between Points 1 and 2; the period *after* parental investment is between Points 2 and 4.

If the sexes are differentially costly, Fisher (1930) predicts that the sex ratio will evolve to favor the cheaper sex. However, there are many potential interpretations of offspring costs. One sex may be costlier because it requires more resources, has greater mortality, or more severely reduces parental survival or reproduction (Charnov 1982; Trivers 1985). Furthermore, these costs may occur at different points in an individual's lifetime (Fig. 4).

We consider four alternative interpretations of offspring costs (summarized in Table 2). For each of these four cases, we will determine how the primary sex ratio *s*_1_ evolves with respect to singular strategy location, evolutionary stability, and convergence stability.

**Table 2:** Summary of four cases where male and female offspring are differentially costly

Cases	Sons have…	Previous predictions	Model results	Example species
0. identical sexes	same costs and vital rates as daughters	*• s*_1_ = 0.5 is a selectively neutral ESS (Fisher 1930; Uyenoyama and Bengtsson 1982; Bull and Charnov 1988)	*• s*_1_ = 0.5 is a selectively neutral ESS (Fig. 5)	*•* explains the prevalence of near 1:1 sex ratios in most species (Hardy 2002)
1. offspring resource cost	greater resource consumption	*• s*_1_ favors the sex that costs fewer resources to produce *• s*_1_ is given by the equal investment principle (1) (Fisher 1930; Charnov 1982; Frank 1990; Hardy 2002)	*• s*_1_ favors the sex that costs fewer resources to produce *•* but *s*_1_ is more biased to the cheaper (more common) sex if couples are poor (Fig. 6)	*•* in wasps, female larvae require larger nest cells (Trivers 1985) or larger hosts (Charnov 1979a) and more food *•* in red deer and elephant seals, males require more milk to wean (Trivers 1985; Frank 1990)
2. offspring mortality cost (*during* investment)	greater mortality and resource consumption	*• s*_1_ favors the higher mortality sex (Fisher 1930; Bodmer and Edwards 1960) *• s*_2_ will be equal or favor the lower mortality sex (Bodmer and Edwards 1960, Merrell 1981; Charnov 1982; West 2009)	*• s*_1_ favors the higher mortality sex (Fig. 8A) *•* but *s*_2_ also favors the higher mortality sex (Fig. 8B)	*•* in many mammals, including humans, males have greater mortality *in utero* (Trivers 1985) *•* rook birds have higher male nestling mortality (Slagsvold et al. 1986)
3. offspring mortality cost (*after* investment)	greater mortality	*• s*_1_ is unaffected by mortality (Fisher 1930; Kolman 1960; Leigh 1970, Charnov 1975; West 2009)	*• s*_1_ is biased toward the lower mortality sex with juvenile mortality (Fig. 9A and B) *• s*_1_ is biased toward the higher mortality sex with adult mortality (Fig. 9C and D)	*•* adult survival is lower for males in both humans (Wisser and Vaupel 2014) and penguins (Jenouvrier et al. 2010)
4. parent mortality cost	greater parental mortality	*• s*_1_ favors the sex that reduces parental survival the least (Charnov 1982)	*• s*_1_ favors the sex that reduces parental survival the least (Fig. 10)	*•* in albatross, male parents with female offspring and low quality female parents with male offspring die more frequently (Weimerskirch et al. 2005) *•* in humans, sons reduce maternal longevity more than daughters (Helle et al. 2002)

1. Offspring resource cost: Different amounts or rates of resources are required to birth male and female offspring (Fig. 4, Point 1). Fisher's sex ratio theory implicitly assumes that parents have a fixed amount of resources for producing offspring (Bull and Charnov 1988). Here, we will assume that total resource investment is always constrained to a constant rate.
2. Offspring mortality cost (*during* investment): Male and female offspring mortality rates differ during the period of parental investment (Fig. 4, Point 1–2); that is, while the offspring is still consuming parental resources. We assume that offspring death during this period frees up resources that can be reallocated to other offspring (Charnov 1982).
3. Offspring mortality cost (*after* investment): Male and female mortality rates differ after the period of parental investment, once the individual is no longer consuming parental resources. We will consider sex‐biased mortality rates for both juveniles (Fig. 4, Point 2–3) and adults (Fig. 4, Point 3–4).
4. Parent mortality cost: Male and female offspring increase the mortality rates of their adult parents (Fig. 4, Point 3–4). For offspring of a given sex, both male and female parents suffer the same mortality increase.

In each case, we will consider a two‐sex, 5‐stage population with the life cycle in Fig. 1. We construct projection matrices of the form (9), adapting the functions and parameters in rate matrices **U** (6), **B** (7), and **T** (8) as necessary to reflect the offspring costs under consideration. The corresponding resident and mutant matrices will be used to calculate the selection gradient (19), from which we can find the SS primary sex ratio $s_{1}^{}*$. We will determine the location and stability properties of $s_{1}^{}*$ in each case. We will also examine the secondary sex ratio *s*_2_ (proportion of adults that are male), focusing specifically on $s_{2}^{}*$ as the value of *s*_2_ when the primary sex ratio *s*_1_ is at its SS value $s_{1}^{}*$.

We make the following assumptions for the underlying two‐sex model:

- Males and females have identical vital rates, save for the offspring cost of interest.
- Only the union stage can produce new offspring. Unmated males and females mate to form unions, but do not reproduce independently.
- Males are always the more “disadvantaged” sex, which is often true in mammals and birds (Table 2). In Case 1, males have higher resource costs. In Cases 2 and 3, male offspring have higher mortality. In Case 4, males impose greater parental mortality. An increase in *s*_1_ thus represents greater production of the disadvantaged sex, while a decrease in *s*_1_ represents increased production of the advantaged sex.

We also make these assumptions for our evolutionary analyses:

- The only evolving trait is the primary sex ratio *s*_1_. Thus, new mutants only differ from established residents in terms of their sex ratio phenotype.
- Mutations are small and do not differ drastically from the resident. They are also rare enough not to affect the resident population, and infrequent enough to either die out or reach fixation before the next mutation arises (Geritz 1996, Metz 2006).
- The mutant phenotype is genetically dominant. Any offspring with a mutant parent also has the mutant phenotype.

Unless otherwise indicated, model parameters are fixed at the values in Table 3. We will consider example parameter sets for two types of unions, “productive” and “poor,” in particular. Productive unions are more persistent (low divorce rate *d*) and can allocate more resources to offspring production (high resource investment rate *R*). Poor unions, in contrast, are more transient (high *d*) and allocate fewer resources to offspring production (low *R*).

**Table 3:** Two‐sex matrix model parameters for productive and poor unions

Parameter	Description	Value (Productive)	Value (Poor)
*μ*_*m*1_, *μ*_*f*1_	male, female juvenile mortality rates	0.1	0.1
*μ*_*m*2_, *μ*_*f*2_	male, female adult mortality rates	0.1	0.1
*α*_*m*_, *α*_*f*_	male, female maturation rates	0.5	0.5
*R*	total resource investment rate	20	10
*d*	union divorce rate	0	1

### Case 0: identically costly sexes

**Figure 5:**
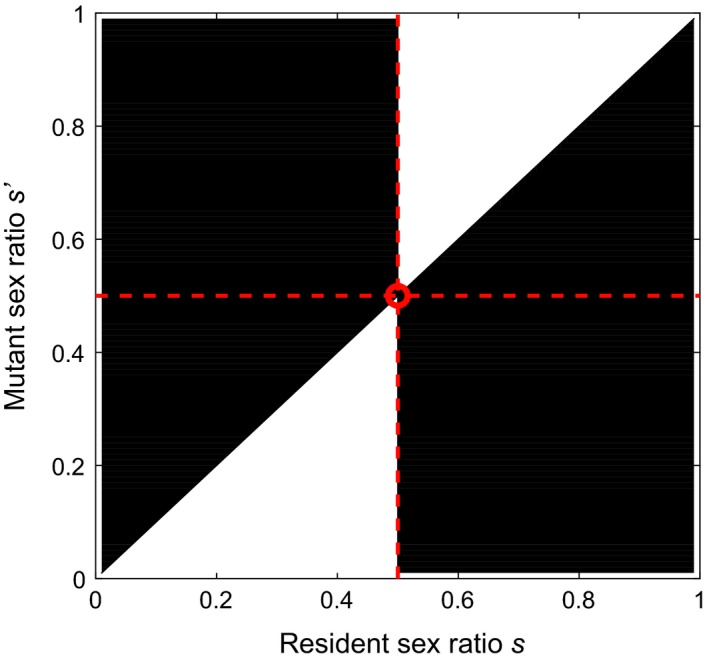
A PIP for sex ratio evolution when males and females are identical. With the parameters in Table 3 (Case 1, productive unions), (22) is 0 and (34) is −0.92 at $s_{1}^{}*=0.5$, confirming that the equal sex ratio is a selectively neutral, convergence stable ESS.

If the sexes are identically costly, selection should favor equal production of males and females. The equal sex ratio is thus an “unbeatable” ESS resistant to invasion by alternative sex ratios (Hamilton 1967; Maynard Smith and Price 1973). Consistent with this classic prediction, our model has a convergent singular strategy at $s_{1}^{}*=0.5$ (example in Fig. 5). This result is robust to other variants of our model, including the various cases of offspring costs, additional stages, different mating functions, etc.

In all cases, the singular strategy intersects with a vertical isocline where mutant and resident growth rates are equal. This is because any mutant that arises when the resident is at the equal sex ratio has an invasion fitness (17) of 0. When $s_{1}^{}*=0.5$, resident males and females are equally abundant at equilibrium. Rare mutants thus have equal mating opportunities with residents regardless of their sex, so all invading sex ratios equally fit. As a result, the equal sex ratio is called a “selectively neutral” strategy (Bull and Charnov 1988) or a “weak form ESS” (Uyenoyama and Bengtsson 1982).

As we shall see in “Evolutionary and convergence stability of the SS sex ratio”, convergence stable, selectively neutral sex ratios like these are the predominant singular strategies in our model.

### Case 1: offspring resource costs

Consider the case where the production of male and female offspring requires different amounts of resources. These production costs are upfront, immediate investments made per birth or conception, and are thus unaffected by later offspring mortality. Parents have a fixed total rate *R* at which they invest resources (energy, food, etc.) into offspring production, so the primary sex ratio *s*_1_ determines how resources are allocated between the sexes.

In this case, we will vary the relative production costs of male and female offspring and determine the ESS sex ratios that result. Assume that producing male offspring requires *C*_*m*_ units of resources per time, while producing female offspring requires *C*_*f*_ units of resources per time. The average resource cost per offspring birth is thus:

(35) $$C_{a}=s_{1}C_{m}+\left(1-s_{1}\right)C_{f}$$

This average offspring cost appears in the birth matrix **B**, as given by (7), and determines how many offspring can be born per time. The union formation matrix **U** and transition matrix **T** are given by (6) and (8).

If demographic structure is ignored, the equal investment principle implies that the primary sex ratio will evolve to favor the cheaper sex. Assume, for example, that *C*_*m*_ + *C*_*f*_ = 1; that is, there is some sort of offspring production trade‐off, so that as males become less costly, females become more costly, and vice versa. Then by (1):

(36) $$s_{1}^{}*=\frac{C_{f}}{C_{m}+C_{f}}=C_{f}$$

The SS sex ratio $s_{1}^{}*$ increases, becoming more male‐biased, as the female cost *C*_*f*_ increases. Similarly, $s_{1}^{}*$ decreases, becoming more female‐biased, as the male cost *C*_*m*_ = 1 − *C*_*f*_ increases.

**Figure 6:**
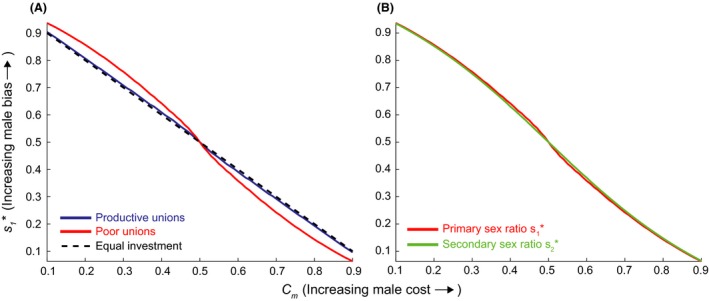
Case 1 singular strategy (SS) sex ratios, as a function of the male offspring cost *C*_*m*_ in (35). In this example, the female offspring cost *C*_*f*_ = 1 − *C*_*m*_. (A) The primary sex ratio $s_{1}^{}*$ for both productive (blue) and poor unions (red). The values of $s_{1}^{}*$ predicted by the equal investment principle (36) are indicated in black. (B) The corresponding secondary sex ratio $s_{2}^{}*$ (green) in the poor unions case.

Consistent with the predictions of the equal investment principle (36), the evolutionarily singular sex ratios in our demographic model are biased toward the cheaper sex (Fig. 6A). For poor unions, however, $s_{1}^{}*$ deviates from the predictions of the equal investment principle in even greater favor of the cheaper sex. This is because the optimal sex ratio depends on a trade‐off between the cost of offspring production (where the cheaper sex is favored because more of it can be produced) and the benefit of offspring reproductive success (where the rarer sex is favored because it has more mating opportunities). However, an increase in mating opportunities is not necessarily proportional to an increase in later births, especially when unions have low reproductive output. When unions are poor, the mating advantage of the rarer, costlier sex is diminished, allowing the trade‐off to skew in favor of the more common, cheaper sex.

Recall that $s_{2}^{}*$ is the secondary sex ratios when $s_{1}=s_{1}^{}*$. In this case, males and females have the same maturation and mortality rates; thus, both $s_{2}^{}*$ and $s_{1}^{}*$ have the same values (Fig. 6B).

### Case 2: offspring mortality *during* parental investment

Rather than paying a single upfront production cost per birth, like in Case 1, parents now pour investments into their offspring over an extended period of time (the period of parental investment shown in Fig. 4, Points 1–2). An offspring's cost (how much parental investment they have consumed) thus accumulates over time, and the cumulative cost of each offspring depends on how long they receive parental investment.

An offspring stops receiving parental investment only when it has reached the age of independence or died. As a result, the expected cost per offspring born depends on the juvenile mortality rates. If more male offspring die before reaching independence, for example, the average cost per male born will be less than that of a female. The average cost per male reared to independence, however, will be higher than that of a female (Fisher 1930).

We will now vary the relative mortality rates of male and female offspring, *during* the period of parental investment, and determine the resulting ESS sex ratios. Assume that males and females have different juvenile mortality rates and will thus have different expected costs per birth. Again, parents invest in offspring at a fixed resource rate *R*. If male offspring have higher mortality rates, the average male born will consume fewer parental resources (and have a lower expected cost) than the average female born.

First, we will determine the expected offspring costs, per male or female born, as a function of the male and female juvenile mortality rates. As in Slagsvold et al. (1986), let *I*(*x*) be the instantaneous parental investment rate in an offspring at age *x*. A parent's cumulative investment *J*(*x*) in that offspring up to age *x* is:

(37) $$J\left(x\right)=\int_{0}^{x}I\left(z\right)dz$$

Let *a* be the age of independence, after which parental investment ceases. If the investment rate is constant so that *I*(*x*) = *I*, (37) becomes:

(38) $$J\left(x\right)=\left\{\begin{array}{cc} Ix, & \text{if}\;x<a\\ Ia, & \text{if}\;x\geq a \end{array}\right.$$

Define *f*(*x*) as the probability that an offspring dies at age *x*. If *μ*(*x*) is the mortality rate at age *x*, it can be shown that (Caswell 2001, Chapter 2):

(39) $$\begin{array}{cc} f(x) & =\mu \left(x\right)e^{-\int_{0}^{x}\mu \left(z\right)dz}\\ & =\mu e^{-\mu x}\;\text{if}\;\mu \;\text{is constant for all}\;x \end{array}$$

The *expected* cumulative investment in an offspring, accounting for its mortality rate during the investment period, is:

(40) $$\begin{array}{cc} E[J(x)] & =\int_{0}^{\infty }J\left(x\right)f\left(x\right)dx\\ & =\int_{0}^{\infty }J\left(x\right)\mu e^{-\mu x}dx\\ & =\int_{0}^{a}J\left(x\right)\mu e^{-\mu x}dx+\int_{a}^{\infty }J\left(x\right)\mu e^{-\mu x}dx\\ & =\int_{0}^{a}Ix\mu e^{-\mu x}dx+\int_{a}^{\infty }Ia\mu e^{-\mu x}dx\\ & =\frac{I}{\mu }\left(1-e^{-\mu a}\right) \end{array}$$

Equation (40) is the expected cost per offspring birth with an offspring mortality rate of *μ*. Male offspring will have a mortality rate *μ*_*m*1_, while female offspring have a mortality rate *μ*_*f*1_. If males and females receive parental investment at the same constant rate *I*, and have ages of independence *a*_*m*_ = 1/*α*_*m*_ and *a*_*f*_ = 1/*α*_*f*_ respectively, the expected male and female offspring costs per birth are thus:

(41) $$\begin{array}{cc} C_{m} & =\frac{I}{\mu_{m1}}\left(1-e^{-\frac{\mu_{m1}}{\alpha_{m}}}\right)\\ C_{f} & =\frac{I}{\mu_{f1}}\left(1-e^{-\frac{\mu_{f1}}{\alpha_{f}}}\right) \end{array}$$

Substitute (41) into (35) to obtain *C*_*a*_, the average offspring cost per birth. Again, the union formation matrix **U**, birth matrix **B**, and transition matrix **T** are given by (6), (7), (8) respectively.

**Figure 7:**
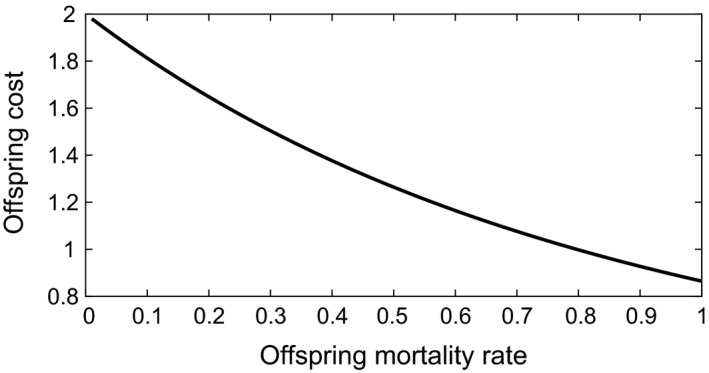
The offspring resource cost (41) as a function of the juvenile mortality rate *μ* (*I* = 1, *α* = 0.5).

Figure 7 shows that the resource cost for a given sex declines as its mortality rate increases, as more and more offspring die before significant parental investment is made. Consider, for example, the case where sons experience greater juvenile mortality than daughters. The average resource cost (41) of each son born is less than that of a daughter, because sons are more likely to die before consuming the full amount of resources needed to reach independence (Bodmer and Edwards 1960, West 2009; Kahn et al. 2015). Based on the equal investment principle (1), we would expect the primary sex ratio to evolve in favor of the higher mortality (lower cost) sex.

**Figure 8:**
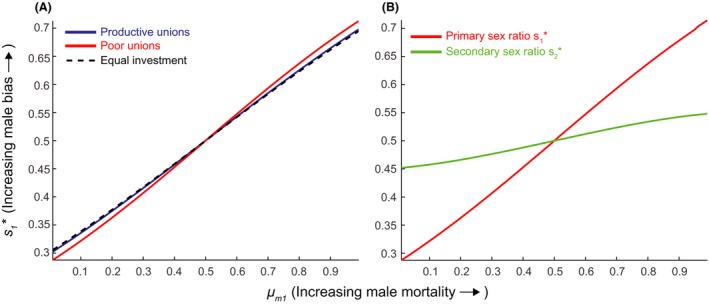
Case 2 singular strategy (SS) sex ratios, as a function of the juvenile male mortality rate *μ*_*m*1_ in (41). We will set the juvenile female mortality rate *μ*_*f*1_ = 1 − *μ*_*m*1_, so that increasing male mortality decreases female mortality and vice versa. (A) The primary sex ratio $s_{1}^{}*$ for both productive (blue) and poor unions (red). The values of $s_{1}^{}*$ predicted by the equal investment principle (1) are indicated in black. (B) The corresponding secondary sex ratio $s_{2}^{}*$ (green) in the poor unions case.

In our model, $s_{1}^{}*$ is indeed biased toward the higher mortality (lower cost) males. As in Case 1, deviations from the equal investment principle increase when unions are poor (Fig. 8A) — again, because the mating advantage of the rarer, costlier sex is seemingly insufficient to compensate for its greater cost. The corresponding secondary sex ratio $s_{2}^{}*$ is less biased than the primary sex ratio, because the cheaper, higher mortality males produced in excess at birth are more likely to die before reaching maturity (Fig. 8B). These results are consistent with the predictions of Fisher (1930), which state that, when the average expenditure is less for each boy born, boys will be more numerous at birth, but less numerous by the end of parental expenditure.

Although $s_{2}^{}*$ is less biased than $s_{1}^{}*$, however, both sex ratios are still biased toward the higher mortality sex. This contradicts previous arguments that the sex ratio should equalize to 0.5 by the age of independence (Bodmer and Edwards 1960) or reproduction (Merrell 1981), or that the sex ratio should favor the lower mortality daughters by the end of parental investment (Charnov 1982). Although the higher mortality sex does become less numerous by maturity (as Fisher originally stated), whether the sex ratio bias equalizes or even reverses is not absolute and likely depends on other factors (e.g., Kahn et al. 2015).

### Case 3: offspring mortality *after* parental investment

Suppose that male and female mortality rates differ *after* the period of parental investment (Fig. 4, Points 2–4). Because sex‐biased risks for disease, competition, selective harvest pressure, etc. can act at any point in the life cycle, we will consider both sex‐biased juvenile mortality (*μ*_*m*1_ ≠ *μ*_*f*1_) and sex‐biased adult mortality (*μ*_*m*2_ ≠ *μ*_*f*2_).

As in Case 2, we will vary the relative mortality rates of male and female offspring, now *after* the period of parental investment, and determine the resulting ESS sex ratios. Assume that male and female offspring have the same resource costs *C*_*m*_ = *C*_*f*_, which we shall normalize to 1. Then, the average offspring cost *C*_*a*_ in (35) is also always 1, and the union reproductive rate *R*/*C*_*a*_ depends only on the (constant) resource investment rate *R*. As offspring mortality does not affect the offspring resource costs, all mortality must occur after the period of investment. Thus, any juvenile mortality in Case 3 occurs in the period between independence and sexual maturity (Fig. 4, Points 2 to 3).

Again, the union formation matrix **U**, birth matrix **B**, and transition matrix **T** given by (6), (7), and (8) respectively. We will fix the stage‐specific mortality rates in **T** at different levels and analyze the sex ratios that evolve.

#### Juvenile mortality

**Figure 9:**
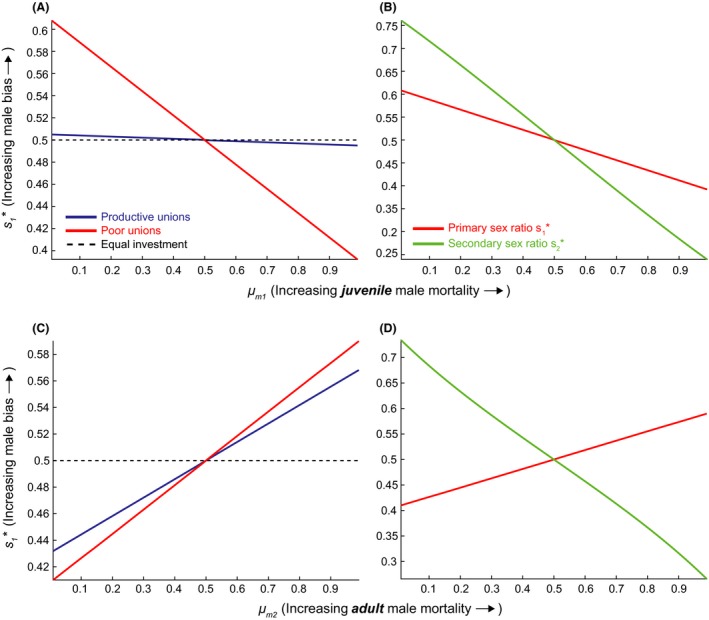
Case 3 singular strategy (SS) sex ratios, as a function of the juvenile male mortality rate *μ*_*m*1_ or adult male mortality rate *μ*_*m*2_ in (8). In the juvenile mortality case, the juvenile female mortality rate *μ*_*f*1_ = 1 ‐ *μ*_*m*1_. In the adult mortality case, the adult female mortality rate *μ*_*f*2_ = 1 − *μ*_*m*2_. (A) The primary sex ratio $s_{1}^{}*$ for both productive (blue) and poor unions (red) in the juvenile mortality case. The values of $s_{1}^{}*$ predicted by the equal investment principle (36) are indicated in black. (B) The corresponding secondary sex ratio $s_{2}^{}*$ (green) in the poor unions, juvenile mortality case. (C) Primary sex ratios for the adult mortality case. (D) Secondary sex ratios for the poor unions, adult mortality case.

Consider the case of sex‐biased juvenile mortality after parental investment. When unions are productive (Fig. 9A, blue), $s_{1}^{}*$ varies slightly as a function of juvenile mortality. This contradicts the predictions of Fisher and many others, who maintain that sex‐biased mortality after parental investment does not affect sex ratio.

When unions are poor (Fig. 9A, red), $s_{1}^{}*$ favors the lower mortality sex even more. This bias occurs for reasons similar to those in Cases 1 and 2. When unions are less productive, the increased mortality of a given sex is not compensated for by its increased mating rates, causing the sex ratio to favor the lower mortality sex.

The secondary sex ratio $s_{2}^{}*$ is even more biased toward the lower mortality sex than the primary sex ratio (Fig. 9B). This is the opposite of Case 2 (mortality occurs during parental investment), where the secondary sex ratio was less biased, but both sex ratios still favored the higher mortality sex. This difference can be explained as follows.

When mortality occurs *during* parental investment (Case 2), the primary sex ratio favors the higher mortality sex. But although more of the higher mortality sex is produced at birth, that sex is also more likely to die before reaching maturity. As a result, both the primary and secondary sex ratios may favor the higher mortality sex, but the secondary sex ratio somewhat *less* so. When mortality occurs *after* parental investment (Case 3, juvenile mortality), the primary sex ratio favors the lower mortality sex. Not only is the lower mortality sex more likely to be produced at birth, but it also has less mortality later on. Thus, both the primary and secondary sex ratios favor the lower mortality sex, the secondary sex ratio somewhat *more* so.

#### Adult mortality

Unlike juvenile mortality, adult mortality expedites union dissolution through the death of mating partners. The return of widows and widowers to the available singles pool subsequently increases mating opportunities for the rarer sex. As a result, $s_{1}^{}*$ actually favors the rarer, higher mortality sex (Fig. 9C), the opposite of the bias in the juvenile mortality case (Fig. 9A).

Once again, these results contradict the Fisherian notion that mortality after parental investment cannot bias the primary sex ratio. As in the case of juvenile mortality, the magnitude of the sex ratio bias is modulated by union productivity. Productive unions have less sex ratio bias, possibly because their larger resource investment rate *R* compensates for unions dissolving due to adult mortality. However, increasing the divorce rate *d* may also reduce sex ratio bias, as the mating advantage of the higher mortality sex is reduced when unions dissolve more easily.

Although the primary sex ratio now favors the higher mortality sex, adult mortality is high enough to skew the secondary sex ratio $s_{2}^{}*$ toward the lower mortality sex (Fig. 9D).

### Case 4: parental mortality

Consider the case where male and female offspring impose different costs on the survival of their parents. As in Case 3, we will assume equal male and female offspring resource costs, so that *C*_*m*_ = *C*_*f*_ = *C*_*a*_ = 1. In Case 4, however, males and females have the same mortality rates and differ instead in the extra mortality costs, *D*_*m*_ and *D*_*f*_, that they impose on their parents.

We now vary the relative mortality costs of male and female offspring, on their adult parents, and determine the resulting ESS sex ratios. We will assume there is a trade‐off between reproduction and survival, so that parents with a greater total cost of reproduction have greater mortality rates. Reproduction costs depend on the per capita mating function *U*_*m*_ from (5), the resource investment rate *R*, the primary sex ratio *s*_1_, and the male and female parental mortality costs *D*_*m*_ and *D*_*f*_.

Let *E*_*m*_ and *E*_*f*_ be the expected cost of reproduction per male parent and per female parent respectively. Then:

(42) $$\begin{array}{cc} E_{m} & =U_{m}R\left[s_{1}D_{m}+\left(1-s_{1}\right)D_{f}\right]\\ E_{f} & =U_{f}R\left[s_{1}D_{m}+\left(1-s_{1}\right)D_{f}\right] \end{array}$$

Note the parental mortality cost imposed per offspring is fixed at birth as *D*_*m*_ or *D*_*f*_, regardless of later offspring mortality. Alternatively, parents could continue incurring mortality costs until their offspring have either died or fully matured.

Because only adults in the union stage *u* produce offspring, adults in the single unmated stages *m*_2_ and *f*_2_ do not experience this extra offspring‐induced mortality. The transition matrix **T** (8) must now distinguish between unmated adult mortality rates (*μ*_*m*2_ and *μ*_*f*2_) and mated adult mortality rates (*μ*_*m*2*c*_ and *μ*_*f*2*c*_).

(43) $$T=\left(\begin{array}{ccccc} -(\mu_{m1}+\alpha_{m}) & 0 & 0 & 0 & 0\\ \alpha_{m} & -\mu_{m2} & 0 & 0 & \mu_{f2c}+d\\ 0 & 0 & -(\mu_{f1}+\alpha_{f}) & 0 & 0\\ 0 & 0 & \alpha_{f} & -\mu_{f2} & \mu_{m2c}+d\\ 0 & 0 & 0 & 0 & -(\mu_{m2c}+\mu_{f2c}+d) \end{array}\right)$$

Only the mated adult mortality rates *μ*_*m*2*c*_ and *μ*_*f*2*c*_ are increased by the costs of reproduction. Let this increase be linearly proportional to the reproductive costs *E*_*m*_ and *E*_*f*_, so that the mortality rates of mated adults are:

(44) $$\begin{array}{cc} \mu_{m2c} & =\mu_{m2}+cE_{m}\\ \mu_{f2c} & =\mu_{f2}+cE_{f} \end{array}$$

where *c* is a nonnegative constant. We will assume that the baseline male and female mortality rates are equal – that is, *μ*_*m*1_ = *μ*_*f*1_ and *μ*_*m*2_ = *μ*_*f*2_ – so the sexes only differ in how they affect parental survival through *D*_*m*_ and *D*_*f*_.

Charnov (1982, Chapter 6) considers a case where mothers experience higher annual mortality rates when having sons instead of daughters. He states that the sex ratio will be biased toward sons so that:

(45) $$\frac{s_{1}}{1-s_{1}}=\frac{\text{maternal mortality for rearing a daughter}}{\text{maternal mortality for rearing a son}}$$

This is equivalent to (1), when offspring costs are framed in terms of a parental mortality expense.

**Figure 10:**
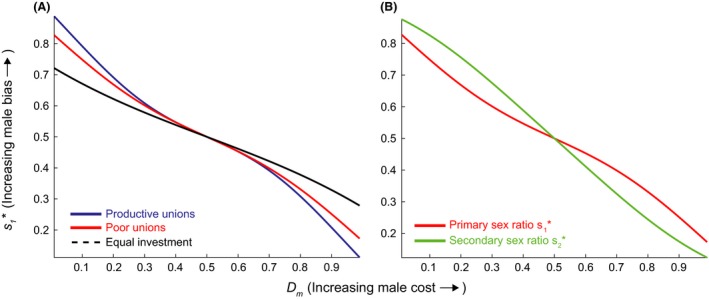
Case 4 singular strategy (SS) sex ratios, as a function of male cost on parental survival *D*_*m*_ in (42), with *c* = 0.1. Wet set the female cost *D*_*f*_ = 1 − *D*_*m*_ (if male offspring impose more parental mortality, female offspring impose less parental mortality, and vice versa). (A) The primary sex ratio $s_{1}^{}*$ for productive unions (blue), poor unions (red), and poor unions with strong juveniles (black). Strong juveniles have lower mortality rates (*μ*_*m*1_ = *μ*_*f*1_ = 0.01) and faster maturation rates (*α*_*m*_ = *α*_*f*_ = 5). (B) The corresponding secondary sex ratio $s_{2}^{}*$ (green) in the poor unions case.

In qualitative agreement with Charvnov's predictions, $s_{1}^{}*$ in our model favors the sex that induces less parental mortality (Fig. 10A). This implies that evolution may favor the preservation of already breeding adults, rather than having them die producing new offspring. Favoring the sex that induces less parental mortality also reduces union dissolution due to partner death.

In contrast to Cases 1–3, productive unions (Fig. 10A, blue) have more sex ratio bias than poor unions – likely because adults with greater reproductive output also have greater parental mortality. However, “strong juveniles” with lower juvenile mortality and higher maturation rates (Fig. 10A, black) reduce the sex ratio bias. In this case, newborn juveniles are faster, more viable replacements for their parents, thereby alleviating the costs of parental death.

Because parental mortality only affects adults, it occurs after the period of parental investment. Thus, as in Case 3, the secondary sex ratio is even more biased toward the cheaper sex than the primary sex ratio is (Fig. 10B).

### Evolutionary and convergence stability of the SS sex ratio

**Figure 11:**
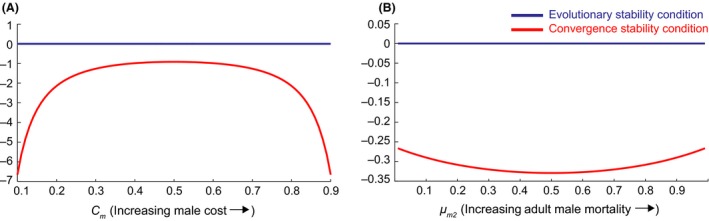
Values of the evolutionary stability condition (22) and convergence stability condition (34) over a range of offspring costs. (A) Case 1, productive unions. (B) Case 3 (adult mortality), poor unions.

The evolutionary and convergence stability properties of the SS sex ratio $s_{1}^{}*$ are identical in Cases 1–4. In all four cases, the second derivative expression (22) is approximately zero and (34) is negative (examples in Fig. 11). Thus, all the singular strategies we have observed are convergence stable “weak form” ESSs, as we previously encountered when male and female offspring were identically costly (Case 0).

As a result, $s_{1}^{}*$ is an evolutionary attractor to which populations will ultimately converge through a series of small mutations. Once the resident population is at $s_{1}^{}*$, any mutant sex ratio will have the same fitness as the resident. However, as the invasion fitness (17) is zero rather than positive, it will not displace the resident through natural selection. Although there is no selection for a new phenotype at a neutral ESS, different rare sex ratios could potentially arise via neutral drift (which may be a mechanism for generating genetic diversity), and even small deviations might shift selective pressures (Bull and Charnov 1988).

The selective neutrality of certain sex ratios has also been noted in models without demographic structure. In Shaw‐Mohler fitness formulation (2), for instance, all individual sex ratios have the same fitness when the population sex ratio is 0.5. Our results suggest that selectively neutral, convergence stable SS sex ratios may be integral features of two‐sex systems in general, as they are maintained even in models with more complex population structure, and consistently appear over a wide range of offspring cost interpretations and values.

### Primary reproductive value ratios

Instead of considering the relative abundances of each sex, as given by the sex ratio, one can also consider their relative reproductive values.

Fisher (1930) originally stated that the total reproductive value of each sex in a given generation must be equal. This notion of reproductive value has been invoked in various ways in studies of sex ratio. Some (e.g., Bodmer and Edwards 1960) specifically consider genetic contributions to grandchildren, so that an individual's reproductive value is inversely proportional to the total surviving individuals of their sex. Others (e.g., Grafen 2014) define an individual's reproductive value as the probability that a random future gene can be traced back to that individual.

We will consider the lifetime reproductive value for each population stage as follows. In a matrix model, the dominant left eigenvector **v** of the projection matrix **A** is a vector of stage‐specific reproductive values (shown in age‐structured models by Goodman 1968; extended to stage‐structured models by Taylor 1990).

Recall that the selection gradient (19) depends on **v**′ as follows:

(46) $$\frac{d\lambda^{\prime }}{ds_{1}^{\prime }}=\left(w^{\prime ⊺}⊗v^{\prime ⊺}\right)\frac{d\text{vec}A^{\prime }}{ds_{1}^{\prime }}$$

For a *s* × 1 population vector, the Kronecker product in (46) is the 1 × *s* ^2^ vector:

(47) $$w^{\prime ⊺}⊗v^{\prime ⊺}=\left(\begin{array}{ccccccccc} w_{1}v_{1} & w_{1}v_{2} & \ldots & w_{1}v_{s} & \left|\ldots \right| & w_{s}v_{1} & w_{s}v_{2} & \ldots & w_{s}v_{s} \end{array}\right)$$

where *w*_*i*_ is the *i* ^th^ entry of **w**′ (stable stage frequency of stage *i*), and *v*_*i*_ is the *i* ^th^ entry of **v**′ (reproductive value of stage *i*).

At any singular strategy sex ratio $s_{1}^{}*$, the selection gradient (46) is equal to 0. Substituting (47) into (46) and evaluating at $s_{1}^{}*$, we obtain:

(48) $$\left(\begin{array}{ccccccccc} w_{1}v_{1} & w_{1}v_{2} & \ldots & w_{1}v_{s} & \left|\ldots \right| & w_{s}v_{1} & w_{s}v_{2} & \ldots & w_{s}v_{s} \end{array}\right)\left(\frac{d\text{vec}A^{\prime }}{ds_{1}^{\prime }}\right){|}_{s_{1}^{\prime }=s_{1}=s_{1}^{*}}=0$$

where

(49) $$\frac{d\text{vec}A^{\prime }}{ds_{1}^{\prime }}=\frac{1}{3}\left(\frac{d\text{vec}T^{\prime }}{ds_{1}^{\prime }}+\frac{d\text{vec}B^{\prime }}{ds_{1}^{\prime }}+\frac{d\text{vec}U^{\prime }}{ds_{1}^{\prime }}\right)$$

#### Cases 1 and 2

In Case 1 (offspring resource costs, **B**′ is a function of $s_{1}^{\prime }$, but **U**′ and **T**′ are not. Thus, (49) simplifies to:

(50) $$\frac{d\text{vec}A^{\prime }}{ds_{1}^{\prime }}=\frac{1}{3}\frac{d\text{vec}B^{\prime }}{ds_{1}^{\prime }}$$

The matrix **B** is given by (7) so that:

(51) $$\begin{array}{cc} \text{vec}B^{\prime } & ={\left(\begin{array}{ccccccc} 0 & \ldots & \frac{Rs_{1}^{\prime }}{C_{a}} & 0 & \frac{R(1-s_{1}^{\prime })}{C_{a}^{\prime }} & 0 & 0 \end{array}\right)}^{⊺}\\ \frac{d\text{vec}B^{\prime }}{ds_{1}^{\prime }} & ={\left(\begin{array}{ccccccc} 0 & \ldots & \frac{RC_{f}}{C_{a}^{2}} & 0 & \frac{-RC_{m}}{C_{a}^{\prime 2}} & 0 & 0 \end{array}\right)}^{⊺} \end{array}$$

where *C*_*a*_ is given by (35).

Substituting (51) into (50), then into (48), we obtain:

(52) $$\frac{C_{f}R}{C_{a}^{2}}w_{s}v_{1}-\frac{C_{m}R}{C_{a}^{2}}w_{s}v_{3}=0$$

Canceling out terms and rearranging, we obtain the simple expression:

(53) $$C_{f}v_{1}=C_{m}v_{3}$$

Again, *v*_*i*_ corresponds to the reproductive value of stage *i*. In our population vector (3), stage 1 is *m*_1_ (juvenile males), and stage 3 is *f*_1_ (juvenile females). Thus, (53) becomes:

(54) $$\frac{v_{m1}}{v_{f1}}=\frac{C_{m}}{C_{f}}$$

The expression (54) shows that, at $s_{1}^{}*$, the primary reproductive value ratio $\frac{v_{m1}}{v_{f1}}$ (ratio of juvenile male to juvenile female reproductive values) equals the ratio of the sex‐specific resource costs. This expression is analogous to the inverse of the equal investment principle (1), but is written in terms of the reproductive value ratio rather than the sex ratio.

The same result (54) holds for Case 2 (offspring mortality *during* parental investment, if *C*_*m*_ and *C*_*f*_ are given by (41).

#### Case 3

In Case 3 (offspring mortality *after* parental investment, (49) once again simplifies to (50). The matrix **B** is given by (7) so that:

(55) $$\begin{array}{cc} \text{vec}B^{\prime } & ={\left(\begin{array}{ccccccc} 0 & \ldots & Rs_{1}^{\prime } & 0 & R(1-s_{1}^{\prime }) & 0 & 0 \end{array}\right)}^{⊺}\\ \frac{d\text{vec}B^{\prime }}{ds_{1}^{\prime }} & ={\left(\begin{array}{ccccccc} 0 & \ldots & R & 0 & -R & 0 & 0 \end{array}\right)}^{⊺} \end{array}$$

Substituting (55) into (50), then into (48), we obtain:

(56) $$Rw_{s}v_{1}-Rw_{s}v_{3}=0$$

which reduces to

(57) $$v_{m1}=v_{f1}$$

In other words, the reproductive values of juvenile males *v*_*m*1_ and juvenile females *v*_*f*1_ are equal at $s_{1}^{}*$. The corresponding primary reproductive value ratio $\frac{v_{m1}}{v_{f1}}$ is thus 0.5 regardless of sex‐specific mortality.

#### Case 4

In Case 4 (parental mortality cost, both **B**′ (15) and **T**′ (43) are functions of $s_{1}^{\prime }$, so (49) becomes:

(58) $$\frac{d\text{vec}A^{\prime }}{ds_{1}^{\prime }}=\frac{1}{3}\left(\frac{d\text{vec}B^{\prime }}{ds_{1}^{\prime }}+\frac{d\text{vec}T^{\prime }}{ds_{1}^{\prime }}\right)$$

After differentiating and performing several algebraic manipulations, (48) yields the expression:

(59) $$v_{m1}=v_{f1}+c\left[\left(D_{f}-D_{m}\right)\left(U_{f}v_{m2}+U_{m}v_{f2}-\left(U_{f}+U_{m}\right)v_{u}\right)\right]$$

In this case, the relationship between reproductive values is more complex. The amount by which *v*_*m*1_ deviates from *v*_*f*1_ is determined by the mortality effect *c*. In the limit as *c* → 0, *v*_*m*1_ → *v*_*f*1_, as in Case 3.

## Discussion

Because reproduction is shaped by the entire life cycle, and stage‐specific offspring costs are often speculated to affect sex ratios, demographic population models lend additional insight into sex ratio theory. We have shown how to formulate flexible demographic two‐sex models, and how to perform evolutionary analyses of these models using adaptive dynamics. Our analyses include calculations and characterizations of singular strategies that depend on sex and stage differences, demonstrating how demographic considerations affect evolution.

Using this approach, we found that four alternative interpretations of sex‐biased offspring costs may modify the primary sex ratio (Table 2). In some cases, our results contradict the widely held conclusions of models that neglect demographic population structure, most notably the classic belief that mortality after the period of parental investment cannot affect the primary sex ratio.

### The importance of union formation

Our results may arise our incorporation of a union stage. Two‐sex models that do not include unions allow adults to reproduce directly and thus do not distinguish between the “mating advantage” and “offspring production advantage” of the rarer (e.g., higher mortality) sex.

**Figure 12:**
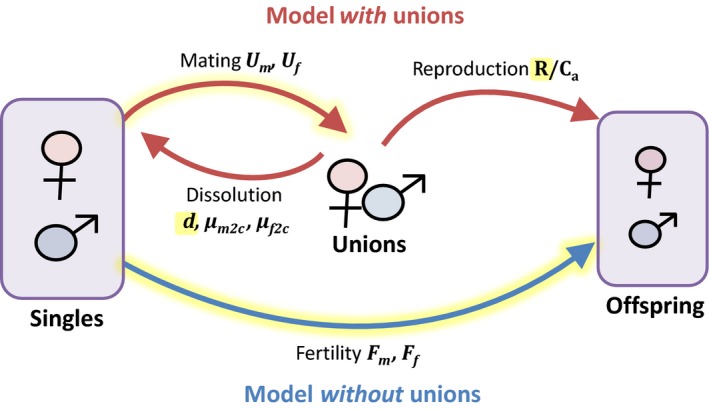
A comparison of two‐sex models with unions (top, red arrows) and without unions (bottom, blue arrow). Parameters used as indicators of union productivity (the divorce rate *d* and resource investment rate *R*) are highlighted in yellow. The highlighted arrows indicate the transitions increased by the “mating advantage” (red highlighted arrow) and “offspring production advantage” (blue highlighted arrow) of the rarer sex.

Figure 12 compares the general structure of models with and without unions. A “mating advantage” increases the rate at which singles form unions (highlighted red arrow), while a “offspring production advantage” (highlighted blue arrow) increases the rate at which singles ultimately produce offspring.

In models *without* unions, single males and females produce offspring directly. In these models, the birth rate is often proportional to the mating function (e.g., Caswell and Weeks 1986; Hardy 2002), so the rarer (higher mortality) sex will have greater fertility and produce more offspring. The directly increased “offspring production advantage” of this higher mortality sex appears to counterbalance its mortality and maintain $s_{1}^{}*$ at equality.

In models *with* unions, singles must first enter the union stage to produce offspring. Single adults enter unions at rates given by the mating functions and may return to the singles stages due to union dissolution from divorce or partner mortality. In this case, the rare, higher mortality sex will have greater mating rates, which may increase its offspring production indirectly. However, this “mating advantage” of the rarer sex is not always proportional to its ultimate “offspring production advantage”. If unions are poor, due to low resource investment rate *R* or high divorce *d*, they ultimately may not produce many offspring. The “offspring production advantage” of the rarer (higher mortality) sex may thus be reduced, causing $s_{1}^{}*$ to favor the more common (lower mortality) sex.

**Figure 13:**
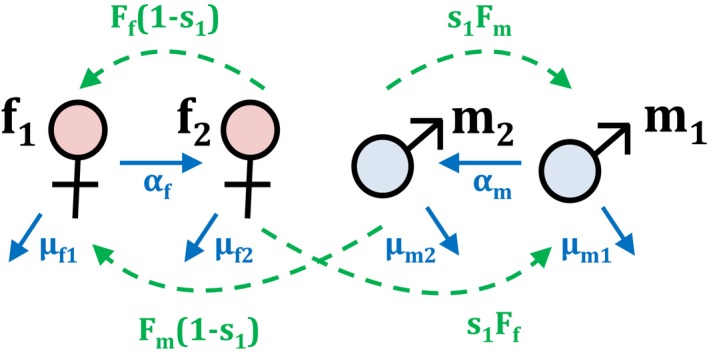
Life cycle diagram for a 4‐stage population with juvenile males *m*_1_ and juvenile females *f*_1_, and adult males *m*_2_ and adult females *f*_2_. The functions and parameters shown here appear in the birth matrix ***bfB*** (64) (green), or transition matrix **T** (65) (blue).

As an illustrative example of a model without unions, consider a 4‐stage model that allows all adults to produce offspring directly. This population contains nonbreeding (juvenile) males *m*_1_ and females *f*_1_ that mature into breeding (adult) males *m*_2_ and females *f*_2_, which then produce new offspring (Fig. 13).

The 4‐stage population vector is:

(60) $$n\left(t\right)=\left(\begin{array}{c} m_{1}\\ m_{2}\\ f_{1}\\ f_{2} \end{array}\right)$$

We now use birth rates rather than mating rates. Assume, as in Case 3, that all offspring resource costs are normalized to 1 (*C*_*m*_ = *C*_*f*_ = *C*_*a*_ = 1). The total birth rate *B*(**n**) is the product of the resource investment rate *R* and the total mating function *M*(**n**) from (4):

(61) B(n)=RM(n)

The corresponding per capita male and female fertility rates are:

(62) $$F_{m}\left(n\right)=\frac{B(n)}{2m}$$

(63) $$F_{f}\left(n\right)=\frac{B(n)}{2f}$$

where the factor of $\frac{1}{2}$ prevents double‐counting offspring from both males and females.

Because we have eliminated the mating process, the mating matrix **U** is simply a matrix of zeros. The birth and transition rate matrices are now:

(64) $$T=\left(\begin{array}{cccc} -(\mu_{m1}+\alpha_{m}) & 0 & 0 & 0\\ \alpha_{m} & -\mu_{m2} & 0 & 0\\ 0 & 0 & -(\mu_{f1}+\alpha_{f}) & 0\\ 0 & 0 & \alpha_{f} & -\mu_{f2} \end{array}\right)$$

(65) $$T=\left(\begin{array}{cccc} -(\mu_{m1}+\alpha_{m}) & 0 & 0 & 0\\ \alpha_{m} & -\mu_{m2} & 0 & 0\\ 0 & 0 & -(\mu_{f1}+\alpha_{f}) & 0\\ 0 & 0 & \alpha_{f} & -\mu_{f2} \end{array}\right)$$

**Figure 14:**
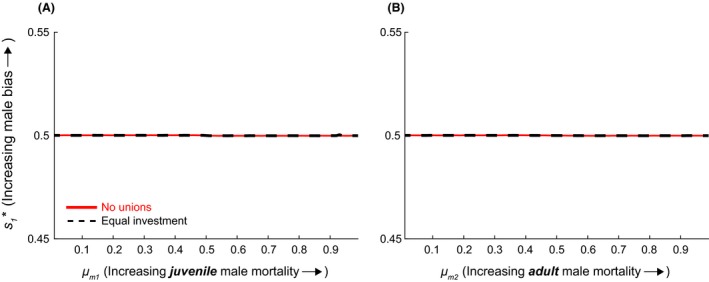
Singular strategy (SS) sex ratios for the 4‐stage (no unions) model, as a function of the (A) juvenile male mortality rate *μ*_*m*1_, with juvenile female mortality *μ*_*f*1_ = 1 ‐ *μ*_*m*1_, or the (B) adult male mortality rate *μ*_*m*2_, with adult female mortality *μ*_*f*2_ = 1 ‐ *μ*_*m*2_.

As in Case 3, we will consider sex‐biased mortality after parental investment. As shown in Figure 14, the equal sex ratio $s_{1}^{}*=0.5$ is now preserved for both juvenile and adult mortality. In the 4‐stage model without unions, higher mortality in one sex appears to be fully compensated for by its higher fertility.

Kahn et al. (2013), who compared models with overlapping generations (like ours) to more typical nonoverlapping generation models, found that sex‐specific adult mortality in the presence of generational overlap can also produce sex ratio biases. In our demographic analyses, however, both models with and without unions have overlapping generations. Because the effect of sex‐biased mortality after parental investment disappears in the no‐union case (Fig. 14), generational overlap alone cannot explain the patterns of sex ratio biases that we have observed.

### The role of reproductive value

**Figure 15:**
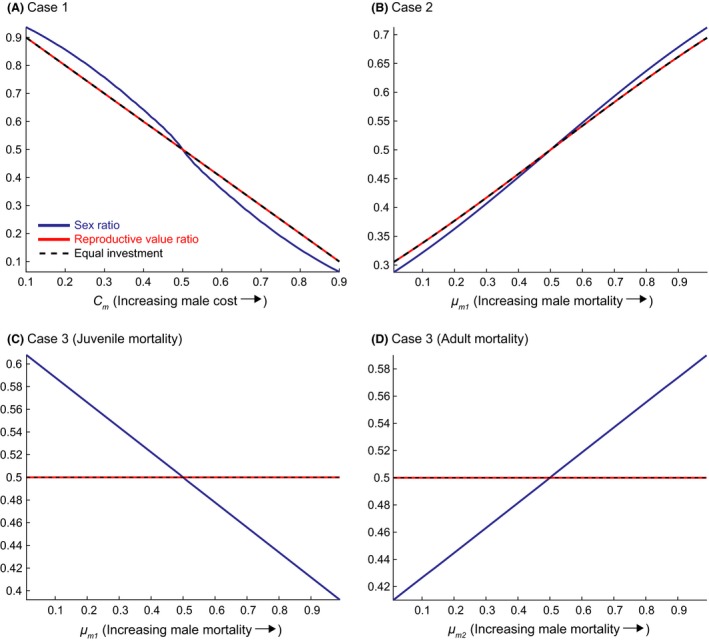
A comparison of the primary sex ratios (blue) and reproductive value ratios (red) with the equal investment principle ratio (1) (black). In (A) Case 1 (offspring resource costs) and (B) Case 2 (offspring mortality *during* parental investment), the primary reproductive value ratio is given by the inverse of (54). In (C) Case 3, juvenile mortality *after* parental investment and (D) Case 3, adult mortality *after* parental investment, the primary reproductive value ratio is always 0.5 by (57). Parameters for poor unions (Table 3) were used in all cases.

We have found that several well‐known predictions about the primary sex ratio are actually more applicable to the primary reproductive value ratio. This includes the equal investment principle (1), and the claim that mortality after parental investment cannot bias the sex ratio. Although we found deviations from the sex ratios predicted by the equal investment principle in Case 1 (offspring resource costs) and Case 2 (offspring mortality *during* parental investment), (54) shows that an analogous principle still holds for the reproductive value ratio instead (Fig. 15A and B). We also found that mortality after parental investment can bias the SS sex ratio (Case 3, offspring mortality *after* parental investment), but it cannot bias the corresponding reproductive value ratio by (57) (Fig. 15C and D).

Consequently, we would only expect the primary sex ratio to follow an equal investment principle and be unaffected by mortality after investment if it were equal to the primary reproductive value ratio – that is, if the lifetime contribution of each sex to future generations was directly proportional to its relative abundance at birth.

However, the primary sex ratio and reproductive value ratios appear to deviate in our 5‐stage model, especially when unions are poor. If unions are unproductive, sex‐specific reproductive values may be differentially reduced, with the rarer sex having much less of a reproductive advantage. The rarer sex must thus become even rarer to raise its reproductive value to the same level as the more common sex, biasing the primary sex ratio in favor of the more common sex.

### Extensions and caveats

We have focused on four common interpretations of sex‐biased offspring costs, but there are many additional sex‐specific differences that can affect the sex ratio, which could studied by the appropriate addition of population stages or rate matrices to our demographic model. For example, male and female offspring may differ not only in how they affect parental survival, but also in how they affect future parental reproduction. Female red deer, for instance, settle closer to their parents than males do, increasing mate competition (Trivers 1985).

Offspring may also benefit their parents through sex‐specific cooperation. In some cooperatively breeding birds, for instance, young males stay with their parents for several years to help rear new broods. This may cause the sex ratio to favor the more “helpful” sex, as evidenced by male‐biased fledgling ratios in woodpeckers (Frank 1990).

We also note that there is often not a single, fixed limiting resource for offspring production (Frank 1990), as we have assumed in Cases 1 and 2. The offspring costs or gains per unit of parental investment are not always fixed as well and may vary according to a nonlinear returns model (Charnov 1979b). These factors may cause additional sex ratio biases that were not noted here. The evolutionary effects of these other considerations, or multiple costs acting simultaneously, could likely be modeled using a similar approach.

## Conflict of Interest

None declared.

## Appendix A Relationship between second derivatives at singular strategies

Following Geritz et al. (1998), we note that *s*_*x*_(*y*) = 0 at any singular strategy *y* = *x* = *x** – that is, if the mutant and resident phenotypes are identical, the invasion fitness is 0. The directional derivative of *s*_*x*_(*y*) along *y* = *x*,* D*_*y*=*x*_[*s*_*x*_(*y*)], is thus also 0:

(A.1) $$\begin{array}{cc} D_{y=x}\left[s_{x}\left(y\right)\right] & =\frac{\partial s_{x}\left(y\right)}{dx}+\frac{\partial s_{x}\left(y\right)}{dy}\\ & =0\;\text{when}\;y=x=x^{}* \end{array}$$

Similarly, the second‐order directional derivative of *s*_*x*_(*y*) along *y* = *x* must also be 0:

(A.2) $$\begin{array}{cc} D_{y=x}\left(D_{y=x}\left[s_{x}\left(y\right)\right]\right){|}_{y=x=x^{}*} & =\frac{\partial }{\partial x}\left(\frac{\partial s_{x}\left(y\right)}{dx}+\frac{\partial s_{x}\left(y\right)}{dy}\right)+\frac{\partial }{\partial y}\left(\frac{\partial s_{x}\left(y\right)}{dx}+\frac{\partial s_{x}\left(y\right)}{dy}\right)\\ & =\frac{\partial^{2}s_{x}\left(y\right)}{\partial y^{2}}+2\frac{\partial^{2}s_{x}\left(y\right)}{\partial x\partial y}+\frac{\partial^{2}s_{x}\left(y\right)}{\partial^{2}x}\\ & =0\;\;\;\;when\;\;y=x=x^{}* \end{array}$$

Assuming that *s*_*x*_(*y*) is twice continuously differentiable, the following relationship holds at any singular strategy *x**:

(A.3) $$\left(\frac{\partial^{2}s_{x}\left(y\right)}{\partial y^{2}}+2\frac{\partial^{2}s_{x}\left(y\right)}{\partial x\partial y}+\frac{\partial^{2}s_{x}\left(y\right)}{\partial^{2}x}\right){|}_{y=x=x^{}*}=0$$

In the two‐sex matrix model, (A.3) becomes (31).

## Appendix B Calculating mixed second derivatives

As in Shyu and Caswell (2014), the pure second derivatives of *λ*′ with respect to ***θ***′ are:

(B.1) $$\frac{\partial^{2}\lambda^{\prime }}{\partial \theta^{\prime }\partial \theta^{\prime ⊺}}=\frac{d}{d\theta^{\prime ⊺}}\text{vec}\left[{\left(\frac{d\lambda^{\prime }}{d\theta^{\prime ⊺}}\right)}^{⊺}\right]$$

The mixed second derivatives of *λ*′ with respect to ***θ***′ and ***θ*** are similarly:

(B.2) $$\frac{\partial^{2}\lambda^{\prime }}{\partial \theta {\partial \theta }^{\prime ⊺}}=\frac{d}{d\theta^{⊺}}\text{vec}\left[{\left(\frac{d\lambda^{\prime }}{d\theta^{\prime ⊺}}\right)}^{⊺}\right]$$

It can be shown (Caswell 2010) that:

(B.3) $$\frac{d\lambda^{\prime }}{d\theta^{\prime ⊺}}={\left(w^{\prime }⊗v^{\prime }\right)}^{⊺}\frac{d\text{vec}A^{\prime }}{d\theta^{\prime ⊺}}$$

Substituting (B.3) into (B.2), we obtain:

(B.4) $$\frac{\partial^{2}\lambda^{\prime }}{\partial \theta \partial \theta^{\prime ⊺}}=\frac{d}{d\theta^{⊺}}\text{vec}\left[{\left(\frac{d\text{vec}A^{\prime }}{d\theta^{\prime ⊺}}\right)}^{⊺}\left(w^{\prime }⊗v^{\prime }\right)\right]$$

To evaluate (B.4), we will use the following rule (Magnus 2010) for derivatives of matrix products. Given matrices **Y** (*n* × *v*) and **X** (*m* × *n*), the derivative of their product with respect to a third matrix **Z** (*p* × *q*) is

(B.5) $$\frac{d\text{vec}(XY)}{d{\left(\text{vec}Z\right)}^{⊺}}=\left(Y^{⊺}⊗I_{m}\right)\frac{d\text{vec}X}{d{\left(\text{vec}Z\right)}^{⊺}}+\left(I_{v}⊗X\right)\frac{d\text{vec}Y}{d{\left(\text{vec}Z\right)}^{⊺}}.$$

Using (B.5), we can rewrite (B.4) as:

(B.6) $$\begin{array}{cc} \frac{\partial^{2}\lambda^{\prime }}{\partial \theta \partial \theta^{\prime ⊺}}= & \;\;\left(w^{\prime ⊺}⊗v^{\prime ⊺}⊗I_{s}\right)\frac{d}{d\theta^{⊺}}\text{vec}\left[{\left(\frac{d\text{vec}A^{\prime }}{d\theta^{\prime ⊺}}\right)}^{⊺}\right]\\ & +{\left(\frac{d\text{vec}A^{\prime }}{d\theta^{\prime ⊺}}\right)}^{⊺}\frac{d\text{vec}(w^{\prime }⊗v^{\prime })}{d\theta^{⊺}}\\ & =\left(w^{\prime ⊺}⊗v^{\prime ⊺}⊗I_{s}\right)\frac{d\text{vec}(C^{⊺})}{d\theta^{⊺}}+C^{⊺}\frac{d\text{vec}(w^{\prime }⊗v^{\prime })}{d\theta^{⊺}} \end{array}$$

where

(B.7) $$C=\frac{d\text{vec}A^{\prime }}{d\theta^{\prime ⊺}}$$

To evaluate the derivative in the first term of (B.6), recall **A**′ is a matrix function of **w**(***θ***).

Thus, to find the matrix derivative of **C** with respect to ***θ***, apply the commutation matrix and chain rule for matrix derivatives:

(B.8) $$\begin{array}{cc} \frac{d\text{vec}(C^{⊺})}{d\theta^{⊺}} & =K_{n^{2},s}\frac{d\text{vec}(C)}{d\theta^{⊺}}\\ & =K_{n^{2},s}\frac{d\text{vec}(C[w(\theta )])}{d\theta^{⊺}}\\ & =K_{n^{2},s}\frac{d\text{vec}C}{dw^{⊺}}\frac{dw}{d\theta^{⊺}} \end{array}$$

where it can be shown (Caswell 2008) that

(B.9) $$\begin{array}{cc} \frac{dw}{d\theta^{⊺}}= & {\left[\lambda I_{s}-A+{\mathrm{we}}^{⊺}A-\left[w^{⊺}⊗\left(I_{s}-{\mathrm{we}}^{⊺}\right)\right]\frac{d\text{vec}A}{dw^{⊺}}\right]}^{-1}\\ & \times \left[w^{⊺}⊗\left(I_{s}-{\mathrm{we}}^{⊺}\right)\right]\frac{d\text{vec}A}{d\theta^{⊺}} \end{array}$$

To evaluate the derivative in the second term of (B.6), we will use the following rule (Magnus and Neudecker 1999, p. 227) for the derivatives of Kronecker products. Given matrices **Y** (*u* × *v*) and **X** (*m* × *n*), the derivative of their Kronecker product with respect to a third matrix **Z** (*p* × *q*) is

(B.10) $$\frac{d\text{vec}(X⊗Y)}{d{\left(\text{vec}Z\right)}^{⊺}}=\left(I_{n}⊗K_{vm}⊗I_{u}\right)\left[\left(I_{mn}⊗\text{vec}Y\right)\frac{d\text{vec}X}{d{\left(\text{vec}Z\right)}^{⊺}}+\left(\text{vec}X⊗I_{uv}\right)\frac{d\text{vec}Y}{d{\left(\text{vec}Z\right)}^{⊺}}\right]$$

Using (B.10), the derivative in the second term of (B.6) becomes:

(B.11) $$\frac{d\text{vec}(w^{\prime }⊗v^{\prime })}{d\theta^{⊺}}=\left(I_{n}⊗v^{\prime }\right)\frac{dw^{\prime }}{d\theta^{⊺}}+\left(w^{\prime }⊗I_{n}\right)\frac{dv^{\prime }}{d\theta^{⊺}}$$

By chain rule,

(B.12) $$\frac{dw^{\prime }}{d\theta^{⊺}}=\frac{dw^{\prime }}{d\text{vec}A^{\prime ⊺}}\frac{d\text{vec}A^{\prime }}{dw^{⊺}}\frac{dw}{d\theta^{⊺}}$$

where $\frac{dw^{\prime }}{d\text{vec}A^{\prime ⊺}}$ is given by (26). Similarly,

(B.13) $$\frac{dv^{\prime }}{d\theta^{⊺}}=\frac{dv^{\prime }}{d\text{vec}A^{\prime ⊺}}\frac{d\text{vec}A^{\prime }}{dw^{⊺}}\frac{dw}{d\theta^{⊺}}$$

where $\frac{dv^{\prime }}{d\text{vec}A^{\prime ⊺}}$ is given by (27).

Substituting (B.8) and (B.11) into (B.6), we obtain:

(B.14) $$\frac{\partial^{2}\lambda^{\prime }}{\partial \theta \partial \theta^{\prime ⊺}}=\left(w^{\prime ⊺}⊗v^{\prime ⊺}⊗I_{s}\right)K_{n^{2},s}\frac{d\text{vec}C}{dw^{⊺}}\frac{dw}{d\theta^{⊺}}+C^{⊺}\left[\left(I_{n}⊗v^{\prime }\right)\frac{dw^{\prime }}{d\theta^{⊺}}+\left(w^{\prime }⊗I_{n}\right)\frac{dv^{\prime }}{d\theta^{⊺}}\right]$$

This expression is equivalent to (32) when the only evolving trait is the primary sex ratio, so that ***θ*** = *s*_1_.
